# Development and Characterization of Novel EST-SSRs from *Larix gmelinii* and Their Cross-Species Transferability

**DOI:** 10.3390/molecules200712469

**Published:** 2015-07-09

**Authors:** Guojun Zhang, Zhenzhen Sun, Di Zhou, Min Xiong, Xian Wang, Junming Yang, Zunzheng Wei

**Affiliations:** 1 Beijing Vegetable Research Center, Beijing Academy of Agriculture and Forestry Sciences, Key Laboratory of Biology and Genetic Improvement of Horticultural Crops (North China), Key Laboratory of Urban Agriculture (North), Ministry of Agriculture, Beijing 10097, China; E-Mails: zhangguojun-8@163.com (G.Z.); zhoudi@nercv.org (D.Z.); xiongminbvrc@163.com (M.X.); wangxianbvrc@163.com (X.W.); 2 College of Horticulture Science and Technology, Hebei Normal University of Science & Technology, Qinhuangdao 066600, China; E-Mail: yjmmail@126.com; 3 State Key Laboratory of Forest Genetics and Tree Breeding, Northeast Forestry University, Harbin 150000, China; 4 Beijing Key Laboratory of Separation and Analysis in Biomedicine and Pharmaceuticals, Beijing Institute of Technology; Beijing 10081, China; E-Mail: sunzz1207@126.com

**Keywords:** *Larix gmelinii*, EST-SSRs, genetic diversity, cross-species transferability

## Abstract

A set of 899 *L. gmelinii* expression sequence tags (ESTs), available at the National Center of Biotechnology Information (NCBI), was employed to address the feasibility on development of simple sequence repeat (SSR) markers for Larch species. Totally, 634 non-redundant unigenes including 145 contigs and 489 singletons were finally identified and mainly involved in biosynthetic, metabolic processes and response to stress according to BLASTX results, gene ontology (GO) categories and Kyoto Encyclopedia of Genes and Genomes (KEGG) maps. Approximately 11.7% (74) unigenes contained 90 candidate SSRs, which were mainly trinucleotides (29, 32.2%) and dinucleotides (26, 28.9%). A relatively high frequency of SSRs was respectively found in the Open Reading Frame (ORF, about 54.4%) and 5′-untranslated region (5′-UTR, 31.2%), while a low frequency was observed in the 3′-untranslated region (3′-UTR, about 14.4%). Of the 45 novel EST-SSRs markers, nine were found to be polymorphic at two *L. gmelinii* populations. The number of alleles per locus (*Na*) ranged from two to four, and the observed (*Ho*) and expected (*He*) heterozygosity values were 0.200–0.733 and 0.408–0.604, respectively. The inbreeding coefficients (*F_IS_*) for all loci were more than zero except Lg41. Most of these 9EST-SSR markers were transferable to its related species *L. kaempferi*, *L. principis-rupprechtii* and *L. olgensis*. These novel EST-SSRs will be useful for further research on comparative genomics, genetic resources conservation and molecular breeding in larch trees.

## 1. Introduction

*Larix* spp., a deciduous coniferous species with a widespread distribution in the world, play vital role in maintaining the structure and functional stability of the ecosystems, and serving wood source and timber industry. In order to accomplish its nature resources conservation and breeding utilization, more recent researches using several molecular markers types were focused on larch’s genetic diversity and population structure [1,2,3,4], genetic relationships and phyletic evolution [5,6,7], species-specific diagnostic [7,8,9] and genetic improvement [10,11,12]. Random dominant molecular markers such as Random Amplification of Polymorphic DNA (RAPD), Inter-Simple Sequence Repeat (ISSR), Amplified Fragment Length Polymorphism (AFLP),except for co-dominant SSR marker, were more extensively employed in the abovementioned previous studies, despite the fact SSR possesses high abundance, stable reproducibility, more ease of use and so on [13]. The main reason is attributed to the limited numbers of developed SSR markers. To date, only less than 100 SSRs including genomic-derived SSRs (gSSRs) and EST-derived SSRs (EST-SSRs) in larch tree were available for effective application [11,14,15,16,17].

Compared with gSSRs, the development of EST-SSRs based on transcribed sequences has become a more fast-acting, cost-effective and labor-saving option for non-model organisms [13]. EST-SSR is present in gene coding regions, which can be identified either by EST database searches or by *in silico* approaches. Two previous reports on larch EST-SSRs development have already achieved relatively favorable gains, especially from pre-existing or open access EST databases. A comprehensive set of 1620 expression sequences, comprised of 313 ESTs from young shoot, xylem and somatic embryos of *L. leptolepis*, and 1147 UniESTs from somatic embryos and a stem Suppression Subtractive Hybridization library (SSH) of larch hybrids (*L. leptolepis* × *L. principis-rupprechtii* and *L. eptolepis* × *L. olgensis*) were first employed to develop six polymorphism EST-SSRs for *L. leptolepis*, and all of them showed high cross-species transferability in the Larix genus [16]. Otherwise, a total of 40,608 Pinaceae ESTs available at the NCBI (http://ww.ncbi.nlm.nih.gov/dbest/) were also downloaded (up to November 2009) and 796 SSR primers were designed to test its cross-genus transferability and polymorphic separate in hybrids population of *L. leptolepis* × *L. gmelinii* [11]. However, only 125 EST-SSRs produced amplification products in the parents (*L. leptolepis* and *L. gmelinii*) and its hybrids. Such a small proportion of successful primer pairs may be attributed to the high levels of complexity and differences among coniferous species genomes. Obvious, more work is needed to identify and develop EST-SSR markers for future research on larch trees.

A set of additional EST sequences of *L. gmelinii* from young needles treated with jasmonic acid (JA) has previously been deposited at NCBI [18], however SSR loci were uncharacterized and not used to develop new EST-SSR markers. Therefore, the objective in present study was to: (1) assemble and annotate these non-redundant sequences; (2) characterize the SSRs motif type and frequency; (3) develop an EST-SSR primer and evaluate its polymorphismin in *L. gmelinii*; (4) test its cross-species transferability in other related larch species.

## 2. Results and Discussion

### 2.1. EST Data Assemble and Annotation

In present study, the available 899 ESTs of *L. gmelinii* were ultimately assembled in 634 unigenes (including 145 contigs and 489 singletons) after trimming and eliminating redundancy. These non-redundant assembly sequences totaled 0.259 Mb and the length of contigs and singlets ranged from 113 to 951 bp, with an average of 409 bp. To explore their potential function, all 634 unigenes were first blast against the NCBI non-redundant sequence database with a threshold value of ≤1.0 × 10^−5^ (Table S1). A total of 446 (70.4%) potential unigenes could be assigned to putative functions or hypothetical proteins while the remainder showed either no hit or non-significant homology, and may thus represent unique components of the *L. gmelinii* expression sequences. Out of the all sequences showing significant homology, the highest proportion of matching sequences were derived from *Populus trichocarpa* (330) and other poplar species (26), members of the Salicaceae family, suggesting that most of EST sequences may correspond to the genes involved in specific woody species growth, development and stress tolerance.

**Figure 1:**
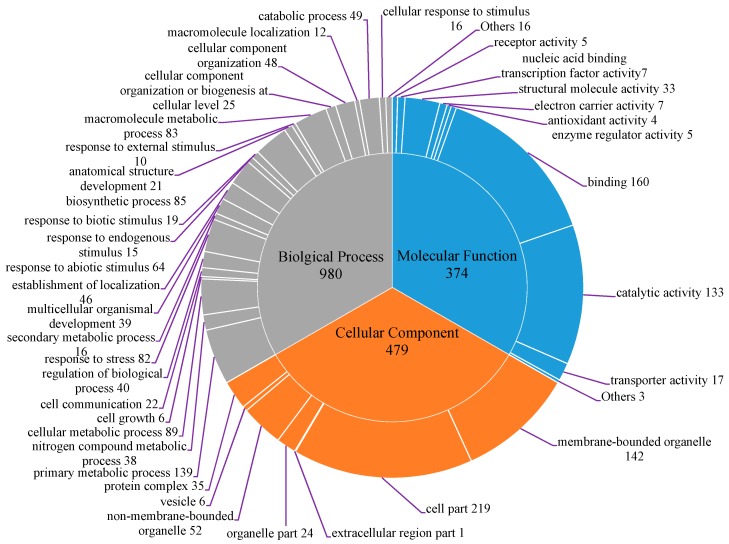
Gene ontology distribution of the *L.* *gmelinii* 634 EST-SSR unigenes into biological process, molecular function and cellular component. The number of transcripts encoded for each category is represented.

Gene ontology categories such as molecular function, biological process and cellular components were then assigned to 446 ESTs unigenes with BLASTX hits using Blast2GO (version 2.6.4, Biobam, Valencia, Spain, http://www.geneontology.org/). A summary for third-level GO term is shown in Figure 1. According to the GO consortium, a total of 980 terms were allocated under biological process, 374 under molecular function and 479 under cellular component. Among the biological processes category, primary metabolic process were the main group (139, 15.2%), followed by cellular metabolic process (89, 9.1%), biosynthetic process (85, 8.7%), macromolecule metabolic process (83, 8.5%) and response to stress (82, 8.4%). For molecular function, the most represented categories were represented by unigenes in the subcategories binding (160, 42.8%) and catalytic activity (133, 35.6%). Meanwhile, a large number of the terms classified as cellular component category were cell part (219, 45.7%), membrane-bounded organelle (142, 29.7%) and non-membrane-bounded organelle (52, 10.9%). Recently, Men *et al.* [19] have performed *de novo* sequencing and assembly of the *L. gmelinii* transcriptome using a short-read sequencing technology (Illumina, San Diego, CA, USA). About 51,157 unigenes were obtained from induced seedlings with JA or methyl jasmonate (MeJA), with a mean length of 517 bp. Apparently, the EST database size in the present study is small or even negligible when compared to the above transcriptome sequences. Nevertheless, the GO classification results were mainly in agreement with reports by Men *et al.* [19]. The high-percentage of unigenes from the categories like binding, metabolic process, biosynthetic process, organelle and cellular process, and a few unigenes from the functions of locomotion, cell killing, virion and virion part [19], demonstrating the assembled EST unigenes in this research has covered the various categories belonging to *L. gmelinii*. In addition, KEGG maps and the EC number were also described in Table S2. In total, 67 KEGG pathways, mainly containing carbohydrate metabolism, methane metabolism, glycolysis/gluconeogenesis and phenylpropanoid biosynthesis, were fully represented by at least one EC number. The phenylpropanoid biosynthesis pathway, also highlighted in research by Men *et al*. [19], plays an important role in plant growth, development and stress resistance. Phenylpropanoids are a group of secondary plant metabolites derived from phenylalanine and function as structural and signaling molecules [19]. Simple phenolic compounds such as aldehydes and alcohols are induced and synthesized via a series of hydroxylation, methylation and dehydration reactions, which are important in plant defense responses against pathogen and herbivore attacks. These annotations will provide a valuable resource for investigating specific defensive processes and genes functions of *L. gmelinii*.

### 2.2. SSR Identification, Motif Frequency Distribution and ORF Prediction

**Figure 2:**
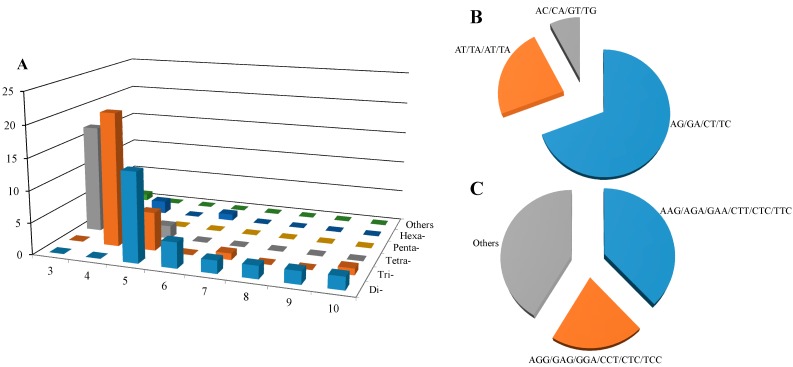
Distribution of SSR motif frequency and typein *L.* *gmelinii* 634 EST-SSR unigenes. (**A**) SSR motif type and repeats numbers; (**B**) Percentage of dinucleotide SSR type; (**C**) Percentage of trinucleotide SSR type.

EST analysis is the most efficient approach, not only for gene discovery, but also for SSR markers identification. In this study, relatively relaxed rules were imposed for SSR loci discovery due to the fewer assembled *L. gmelinii* unigenes. The search for SSRs in the 634 non-redundant EST sequences revealed 90 candidate SSRs in 74 ESTs (25 SSRs derived from 21 contigs and 65 from 54 singletons), suggesting approximately an average frequency of one SSR per 2.87 kb and per 7.04 unique unigenes. In terms of numbers of SSRs per EST, 61 ESTs contained only one SSR while 11, one and one EST had two, three and four SSRs, respectively. The identified 90 candidate SSRs (Figure 2) are comprised of 26 di-, 29 tri-, 21 tetra-, seven penta-, six hexa- and one heptanucleotide. The frequency of trinucleotide motifs with four repeats was more common (21, 23.3%), followed by tetranucleotides of three repeats (17, 18.9%) and dinucleotides of five (14, 15.6%). Among the dinucleotide repeats, the AG/GA/CT/TC motifs showed the most frequency (18, 69.2%), followed by the AT/TA/AT/TA motifs (6, 23.1%) Among the trinucleotide repeats, AAG/AGA/GAA/CTT/CTC/TTC motifs were the most common, accounting for 37.9% (11), followed by AGG/GAG/GGA/CCT/CTC/TCC (6, 20.7%). Other motifs were identified in insignificant numbers. These characterizations of SSR motif distribution, frequency and type are basically consistent with observation in *L. kaempferi* [20] and other than Pinaceae trees species [11], despite the dependence on search criteria, the size of the dataset and the database mining tools [21,22]. However, we should also note that the SSR estimates in the present study may be inflated due to the relaxed search parameters used. Those loci with low numbers of repeats that were found using extremely low thresholds may be non-real SSRs and require further validation. The SSRs’ location in ESTs may suggest their potential role in gene expression and functions. Hence, the position of all EST-SSRs was also estimated in relation to the ORF of the corresponding unigenes. From our dataset, 54.4% (49) of the SSR sequences were within ORF sequences while 31.2% (28) and 14.4% (13) were in 5′-UTR and 3′-UTR respectively. The observation was not in accord with the report on yellow catfish (*Pelteobagrus fulvidraco*) transcriptome sequences, of which 45.3% SSR motifs were present in UTRs and 27.9% in ORF [23]. This difference may be attributed to the quantity and quality of assembled unigenes and species.

### 2.3. EST-SSR Marker Development, Validation and Transferability Analysis in L. gmelinii and Its Related Larch Species

All 74 SSR-containing sequences were used to design primers. Only 45 (60.8%) primer pairs (partial results are shown in Table 1) could be successfully designed for further PCR optimization and amplification in larch tree species. The remaining sequences were abandoned because of their poor quality for primer designing criteria, like very short DNA sequences flanking the SSR (at least on one side) or inappropriate flanking sequences. Using genomic DNAs of ten *L. gmelinii* samples, 28 primer pairs exhibited stable and repeatable amplification after optimization. The followed screening showed that nine were polymorphic across 30 individuals from two populations of *L.* *gmelinii*. Detailed information and population genetic parameters regarding these nine novel polymorphic EST-SSR primers are shown in Table 1. Out of the nine polymorphic EST-SSRs, four are homologous to genes involved in ribosomal and cell wall development (Lg25 and Lg41), pH concentration regulation (Lg14) as well as aromatic amino acids synthesis (Lg32). EST-SSRs variations in these genes may represent their potential role in functional differentiation or adaptation evolution [13,19], which will be useful for diversity studies, cross-species transferability and breeding in larch species.

Among two *L.* *gmelinii* populations, the number of alleles detected at each EST-SSR locus ranged from two (Lg06, Lg14 and Lg36) to four (Lg01 and Lg02), with an average number of 2.60 per locus. The allele range in present study is two to four, which is similar to that of previous studies on *L. kaempferi* using EST-SSRs (2–4) [16], but lower than the number of gSSR markers in *L. kaempferi* (5–26) [14], *L. occidentalis* (5–19) [15] and *L. deciduas* (9–36) [17]. In general, EST-SSR primers are less polymorphic than genomic SSR in plants because of high conservation in transcribed regions [13]. Moreover, other factors [21,22] such as species, populations, sample size and SSR motif type, *etc.* also may be attributed to the difference between EST-SSR and gSSR loci. The observed and expected heterozygosities (*Ho* and *He*) ranged from 0.200 to 0.733 and from 0.278 to 0.704, with the average of 0.411 and 0.489, respectively. No locus except for Lg41 deviated significantly from Hardy–Weinberg equilibrium (HWE). The inbreeding coefficient ranged from −0.150 (Lg41) to 0.464 (Lg06). Eight of nine loci have positive *F_IS_* values (all *p* > 0.05), which indicate a serious heterozygous genotype deficiency. This conclusion is similar to that of other studies on natural *L. gmelinii* populations using RAPD, ISSR and EST-SSR markers [1,2,3,4,5]. In fact, Oreshkova *et al.* [1] have pointed out that a high level of inbreeding is typical for larix species, which could result from its some specific traits like air bag absence in pollen, severe natural and climatic conditions, and pyrogenic influence on its progeny. In additional, other factors like null-alleles or/and population substructure also maybe potential reasons resulting in significant positive *F_IS_* values. Thus more extensive works need to confirm above assumption in further research.

Cross-species amplification was further examined in three species *L. kaempferi*, *L. principis-rupprechtii* and *L. olgensis*, which are also indigenous and important Larch trees in North China (Table 2). Most of the primer pairs amplified comparable products sizes with these three closely related larch species. At these polymorphic loci, *Na* ranged from 1 to 3, while *Ho* and *He* separately ranged from 0–1.000 and 0.08–0.537. However, more loci like Lg01, Lg06 and Lg32 deviated significantly from HWE, which may be resulting from the small size and stringently artificial selection on these populations. The EST-SSR transferable results in the *Larix* genus are in agreement with report by Yang *et al.* [16,20], who verifiedall 6 EST-SSR developed from *L. kaempferi* could be successfully amplified over four species including *L.* *gmelinii*, *L. principis-rupprechtii*, *L. olgensis* and *L. olgensis* var *Koreana*. These high cross-species amplification of EST-SSR demonstrated that the SSR containing sequences or unigenes were extremely conserved in related *Larix* species native to China.

**Table 1:** Characteristics of 10 polymorphic EST-SSR markers from *L.* *gmelinii* unigenes.

Locus	Primers Sequence (5′-3′)	Repeats Motif	Size Range	Ta (°C)	SSR Locations	BLAST Top Hit Accession No.	Description of Putative Function	*E-Value*
Lg01	F: CAGTGGTGTCCGTGGTGTA R:GACCTCCTCCACACCTAAT	(AGC)_4_	141–160	51.3	ORF	XP_006375910.1	hypothetical protein POPTR_0013s05890g (*Populus trichocarpa*)	2.00 × 10^−20^
Lg02	F: CTCTGTGACCAAGAAACCAA R:CATGAAGACGAAGAATGCACT	(AGG)_4_	120–140	51.8	ORF	XP_002306980.2	hypothetical protein POPTR_0005s27390g (*P. trichocarpa*)	3.00 × 10^−38^
Lg06	F: CAAGGATGGAGCAGACGAT R:AGCCTCGCACTTTGACAGA	(AGA)_5_	135–150	50.4	ORF	None	None	None
Lg14	F: GGGGATTGCAGAGTAGAAA R:AAACAGCCATCGAAATGAG	(TC)_6_	140–150	49.5	5′UTR	XP_002319953.1	Xyloglucan endotransglucosylase/hydrolase protein 9 precursor (*P. trichocarpa*)	1.00 × 10^−164^
Lg25	F: GTGAGAGGTCAAACCCCAA R: AGAAGAGTCTGGTCCACGCT	(AAG)_4_	105–125	53.8	ORF	XP_003608708.1	40S ribosomal protein S30 (*Medicago truncatula*)	2.00 × 10^−24^
Lg32	F: CTCTGTCGCACCAGCATTG R:TTGTCTTCCGGTATTCACA	(AT)_6_	105–115	47.5	ORF	XP_002307364.1	2-dehydro-3-deoxyphosphoheptonate aldolase family protein (*P. trichocarpa*)	4.00 × 10^−7^
Lg36	F: TGCCCATCCTCTTTGTTTA R: AGCACCTGATTCCACATTCT	(GA)_5_	175–190	50.6	ORF	None	None	None
Lg37	F: ACAATGGCTTCCTTCAACA R: TATGAGGTGGTTAGGGAGA	(CT)_6_	160–170	47.1	ORF	XP_002299125.2	hypothetical protein POPTR_0001s04570g (*P. trichocarpa*)	3.00 × 10^−46^
Lg41	F: ACTTCCACTAAGGTTGACA R:ATCCACTGCCTTCTGGTCAT	(AGA)_4_	147–180	49.4	ORF	XP_002313280.1	60S ribosomal protein L6 (*P. trichocarpa*)	2.00 × 10^−49^

**Table 2:** EST-SSR Genetic diversity for two populations of *L.* *gmelinii* and its cross-species transferability for *L. kaempferi*, *L. principis-rupprechtii* and *L. olgensis*.

Locus	*L.* *gmelinii* Jiamusi Population			*L.* *gmelinii* Hulunbeier Population			*L. kaempferi*			*L. principis-rupprechtii*			*L. olgensis*		
	(*N* = 15)			(*N* = 15)			(*N* = 8)			(*N* = 10)			(*N* = 9)		
	*Na*	*Ho/He*	*F_IS_*	*Na*	*Ho/He*	*F_IS_*	*Na*	*Ho/He*	*F_IS_*	*Na*	*HoHe*	*F_IS_*	*Na*	*Ho/He*	*F_IS_*
Lg01	2	0.467/0.704	0.338	2	0.200/0.278	0.280	3	0.000/0.406	1.000 **	2	0.100/0.375	0.733 *	2	0.111/0.500	0.778 *
Lg02	2	0.600/0.464	−0.292	4	0.733/0.560	−0.310	2	0.500/0.469	−0.067	3	0.800/0.535	−0.495	2	0.667/0.494	−0.350
Lg06	2	0.267/0.498	0.464	2	0.267/0.498	0.464	2	0.000/0.219	1.000 **	2	0.000/0.180	1.000 **	2	0.000/0.346	1.000 **
Lg14	2	0.200/0.358	0.441	2	0.267/0.480	0.444	1	0.000/0.000	--	2	0.100/0.375	0.733 *	2	0.111/0.278	0.600
Lg25	3	0.400/0.371	−0.078	3	0.733/0.598	−0.227	2	0.250/0.375	0.333	3	0.400/0.465	0.140	2	0.111/0.278	0.600
Lg32	2	0.267/0.444	0.400	3	0.467/0.558	0.163	1	0.000/0.000	--	2	0.000/0.180	1.000 **	2	0.000/0.198	1.000 **
Lg36	2	0.267/0.498	0.464	2	0.267/0.320	0.167	1	0.000/0.000	--	2	0.100/0.255	0.608	2	0.333/0.401	0.169
Lg37	2	0.200/0.358	0.441	3	0.400/0.611	0.345	1	0.000/0.000	--	2	0.100/0.255	0.608	1	0.000/0.000	--
Lg41	3	0.467/0.584	−0.167 *	3	0.600/0.620	−0.150 *	3	0.625/0.617	−0.013	2	0.500/0.495	−0.010	2	0.333/0.500	0.333 *

**: *p* ˂ 0.01; *: *p* ˂ 0.05; --: No available data.

## 3. Experimental Section

### 3.1. Plant Materials

Two *L. gmelinii* populations, separately sampled from Jiamusi in Heilongjiang Province (*N* = 15) and Hulunbeier (*N* = 15) in Inner Mongolia Autonomous Region, were used to screen polymorphism EST-SSR loci. Three other larch populations including one *L. kaempferi* (*N* = 8), one *L. principis-rupprechtii* (*N* = 10) and one *L. olgensis* (*N* = 9), sampled from the National Larch Breeding Base at Weichang in Hebei Province, were used to assess polymorphic SSR cross-species transferability. All collected larch needles were preserved with dry ice and transported to the laboratory. Total genomic DNA extraction was performed using the DNAeasy Plant Mini Kit™ (Tiangen Biotech, Beijing, China), following the manufacturers protocol. The amount and quality of the DNA were evaluated on 1.0% agarose gels prior to subsequent analysis.

### 3.2. EST Data Assemble and Annotation

A total of 899 ESTs of *L. gmelinii* under treated with JA were retrieved from the NCBI and assembled into non-redundant consistent ESTs using SeqMan DNAStar Lasergene (version 7.1, DNASTAR, Madison, WI, USA). To assess the putative identities of the above EST unigenes, BLASTX searches were performed against the GenBank non-redundant protein database (http://blast.ncbi.nlm.nih.gov/Blast.cgi) with an *E*-value cut off 1.0 × 10^−5^. Moreover, Blast2Go was used to assign GO terms, KEGG maps and enzyme classification number (EC number).

### 3.3. SSR Identification and Primer Designs

All SSR loci were identified from the non-redundant EST sequences trimmed by MIcroSAtellite (MISA, version 1.0, Thomas Thiel, Gatersleben, Germany) SSRs were defined as at least five, four, and three motif repeat units for di-, tri- and higher-order nucleotides, respectively. Furthermore, the European Molecular Biology Open Software Suite get ORF (Gary Williams, Cambridge, UK) were used to predict whether the SSR motifs were located within an open reading frame (ORF) [24]. Finally, the primer pairs flanking the region of SSRs were designed with PRIMER5.0 software (version 5.0, PRIMER Biosoft, Palo Alto, CA, USA) with major primer design parameters as follows: product length 100–300 bp, primer size 18–25 bp, and melting temperature between 40 °C and 60 °C.

### 3.4. PCR Amplification, Cross-Species Transferability and Data Analysis

Randomly selected 10 *L. gmelinii* individuals were initially used to test synthesized EST-SSR loci before further population analysis. All PCR amplifications were performed on the Bio-Gener GT9612 system (Baiheng Biotech, Hangzhou, China) in 15 μL reaction volumes containing 1.5 μL 10 × PCR buffer, 30–40 ng template DNA, 1.2 mM MgCl_2_, 0.8 μL dNTPs (2.5 mM each), 0.4 μL each primer (10 μM) and 0.25 unit TaqDNA polymerase (Zexing Biotech, Beijing, China). The thermal profile used for amplifications consisted of 5 min of initial denaturation at 95 °C, followed by 25 cycles of 30 s at 95 °C, 45 s of annealing at the optimized Ta (Table 1), 60 s of extension at 72 °C, and a final extension of 20 min at 72 °C. The DNA amplification products were loaded on denaturing 8.0% polyacrylamide gels. Finally, the gels were silver-stained according to the protocol described by Guan [11]. Fragment sizes were estimated using the 34-501-bp pUC19/Msp I DNA marker (Zexing Biotech, Beijing, China). Descriptive genetic diversity statistics, including *Na* (the number of alleles per locus), *Ho* and *He* (the observedand expected heterozygosity) and *F_IS_* (the inbreeding coefficient) were estimated using GenAlex (version 6.2, Peakall and Smouse, Canberra, Australia).

## 4. Conclusions

Currently, the development of functional molecular markers like EST-SSRs is becoming a valuable objective for plants, especially in marker-assisted breeding programs. However, the number of EST-SSR for larch species is low due to the limited genetic information that is publicly accessible. In this research, a set of EST-SSRs, derived from unexplored *L.* *gmelinii* EST sequences available from the NCBI database, were newly developed and characterized. A total of nine polymorphic EST-SSR markers were ultimately screened and proved across two *L. gmelinii* populations, and were also showed to be highly transferable in related three species, including *L. kaempferi*, *L. principis-rupprechtii* and *L. olgensis*. There is no doubt that these novel EST-SSRs will be helpful for future research on genetic diversity, population structure, evolutionary processes, linkage map construction and QTL mapping in larch trees. As well, it is apparent that the number of EST-SSRs is not much, but this situation will be substantially changed with the application of next-generation sequencing (NGS) technology. At present, large-scale transcript sequences for *L. gmelinii* and *L. kaempferi* [19,25], have been generated and deposited in the public NCBI SRA database, which can be easily employed to develop functional SSR, Single nucleotide polymorphism (SNP) and Insertion/deletion event (InDel) molecular markers at the genome scale in future studies.

## Supplementary Materials

Supplementary materials can be accessed at: http://www.mdpi.com/1420-3049/20/07/12469/s1.
