# Supplementary material for: Development and Characterization of Novel EST-SSRs from Larix gmelinii and Their Cross-Species Transferability
Source: Molecules. 2015 Jul 9;20(7):12469–80. doi: 10.3390/molecules200712469 (PMC6332378; doi:10.3390/molecules200712469)
Supplement: Supplementary file 1 [file molecules-20-12469-s001.pdf]

Supplementary Materials

Table S1. Non-redundant annotation of *L. gmelinii* 634 unigenes in with a threshold value of ≤1.0×10^{-5}.

Sequence	Top Hit Accession No.	Description of Putative Function	*E-Value*
Contig_1	YP_173374.1	hypothetical protein NitaMp027 [*Nicotiana tabacum*]	2.00×10^{-49}
Contig_2	ADW80607.1	sucrose synthase 2 [*Populus tomentosa*]	1.00×10^{-34}
Contig_3	XP_002320450.2	hypothetical protein POPTR_0014s14740g [*Populus trichocarpa*]	2.00×10^{-37}
Contig_5	XP_002305397.1	40S ribosomal protein S19 [*Populus trichocarpa*]	2.00×10^{-98}
Contig_6	XP_002319276.2	zinc finger family protein [*Populus trichocarpa*]	3.00×10^{-66}
Contig_8	AFR41232.1	phenylalanine ammonia-lyase [*Populus nigra*]	5.00×10^{-47}
Contig_9	ABK96039.1	Select seq gb|ABK96039.1| unknown [*Populus trichocarpa*]	6.00×10^{-41}
Contig_10	AGX27222.1	cellulose synthase 7 [*Populus tomentosa*]	5.00×10^{-63}
Contig_11	XP_002325493.1	hypothetical protein POPTR_0019s08760g [*Populus trichocarpa*]	4.00×10^{-32}
Contig_14	XP_002489102.1	hypothetical protein SORBIDRAFT_0070s002020 [*Sorghum bicolor*]	4.00×10^{-06}
Contig_15	XP_003599574.1	hypothetical protein MTR_3g035620 [*Medicago truncatula*]	1.00×10^{-29}
Contig_28	XP_006375910.1	hypothetical protein POPTR_0013s05890g [*Populus trichocarpa*]	2.00×10^{-20}
Contig_30	XP_002306980.2	hypothetical protein POPTR_0005s27390g [*Populus trichocarpa*]	3.00×10^{-38}
Contig_31	XP_006371546.1	hypothetical protein POPTR_0019s13040g [*Populus trichocarpa*]	2.00×10^{-57}
Contig_34	BAD07869.1	hypothetical protein [*Oryza sativa Japonica Group*]	9.00×10^{-07}
Contig_51	ABK94904.1	unknown [*Populus trichocarpa*]	1.00×10^{-25}
Contig_59	AAT37947.1	fasciclin-like AGP 4 [*Populus tremula x Populus alba*]	1.00×10^{-92}
Contig_60	XP_006381469.1	fasciclin-like AGP 13 family protein [*Populus trichocarpa*]	9.00×10^{-101}
Contig_61	AAT37947.1	fasciclin-like AGP 4 [*Populus tremula* × *Populus alba*]	9.00×10^{-05}
Contig_64	XP_002312132.1	18.2 kDa class I heat shock family protein [*Populus trichocarpa*]	2.00×10^{-81}
Contig_65	XP_002308537.1	heat shock protein 17.7 [Populus trichocarpa]	6.00×10^{-27}
Contig_66	XP_006384657.1	17.6 kDa class I small heat shock family protein [*Populus trichocarpa*]	3.00×10^{-84}
Contig_68	XP_002302025.1	isoflavone reductase family protein [*Populus trichocarpa*]	3.00×10^{-65}
Contig_69	ADR31603.1	caffeic acid 5-hydroxyferulic acid 35-*O*-methyltransferase ferulic acid complex chain A [*Populus trichocarpa*]	5.00×10^{-88}
Contig_70	XP_002322264.2	hypothetical protein POPTR_0015s10940g [*Populus trichocarpa*]	3.00×10^{-127}
Contig_71	ABS12346.1	dehydrin [*Populus maximowiczii*]	2.00×10^{-27}
Contig_72	XP_002321064.1	hypothetical protein POPTR_0014s13600g [*Populus trichocarpa*]	4.00×10^{-132}

Table S1. *Cont.*

Sequence	Top Hit Accession No.	Description of Putative Function	*E-Value*
Contig_77	XP_002307384.1	hypothetical protein POPTR_0005s17010g [*Populus trichocarpa*]	4.00×10^{-26}
Contig_78	BAA34919.1	heat shock protein 70 cognate [*Salix gilgiana*]	2.00×10^{-100}
Contig_79	XP_002316294.1	heat shock protein 70 [*Populus trichocarpa*]	4.00×10^{-20}
Contig_80	ABK92609.1	unknown [*Populus trichocarpa*]	4.00×10^{-114}
Contig_82	KDO36164.1	hypothetical protein CISIN_1g024250mg [*Citrus sinensis*]	2.00×10^{-63}
Contig_83	ABK93781.1	unknown [*Populus trichocarpa*]	1.00×10^{-66}
Contig_85	XP_002313519.2	fasciclin-like AGP 15 family protein [*Populus trichocarpa*]	1.00×10^{-31}
Contig_89	XP_002304225.1	Profilin 3 family protein [*Populus trichocarpa*]	3.00×10^{-85}
Contig_90	XP_002308591.1	profilin 2 family protein [*Populus trichocarpa*]	8.00×10^{-73}
Contig_93	XP_002300962.1	1-aminocyclopropane-1-carboxylate oxidase family protein [*Populus trichocarpa*]	2.00×10^{-84}
Contig_95	AFR42184.1	β-tubulin [*Populus trichocarpa*]	5.00×10^{-26}
Contig_97	NP_001235900.1	polyubiquitin [*Glycine max*]	3.00×10^{-33}
Contig_98	NP_001190681.1	ubiquitin 11 [*Arabidopsis thaliana*]	1.00×10^{-97}
Contig_100	XP_006381397.1	*S*-adenosylmethionine synthetase family protein [*Populus trichocarpa*]	7.00×10^{-31}
Contig_104	AGU99972.1	HSP90 [*Populus tomentosa*]	4.00×10^{-14}
Contig_105	AAN77149.1	fiber protein Fb9 [*Gossypium barbadense*]	2.00×10^{-19}
Contig_107	XP_006385374.1	Calcineurin B-like protein 1 [*Populus trichocarpa*]	5.00×10^{-105}
Contig_108	XP_002311382.2	hypothetical protein POPTR_0008s10450g [*Populus trichocarpa*]	5.00×10^{-90}
Contig_109	ABB88893.1	UDP-glucose pyrophosphorylase [*Populus tremula* × *Populus tremuloides*]	2.00×10^{-47}
Contig_110	XP_002316022.1	class1 chitinase family protein [*Populus trichocarpa*]	5.00×10^{-27}
Contig_111G	XP_002317608.1	glutathione transferase family protein [*Populus trichocarpa*]	8.00×10^{-43}
Contig_112	XP_002298729.1	caffeoyl-CoA O-methyltransferase family protein [*Populus trichocarpa*]	3.00×10^{-43}
Contig_113	XP_006381780.1	fructose-1 family protein [*Populus trichocarpa*]	5.00×10^{-107}
Contig_115	XP_006368635.1	hypothetical protein POPTR_0001s06710g [*Populus trichocarpa*]	5.00×10^{-82}
Contig_117	XP_002318014.1	senescence-associated family protein [*Populus trichocarpa*]	1.00×10^{-55}
Contig_119	ABK94636.1	unknown [*Populus trichocarpa*]	1.00×10^{-32}
Contig_123	XP_002318967.2	hypothetical protein POPTR_0013s01230g [*Populus trichocarpa*]	5.00×10^{-88}

Table S1. *Cont.*

**Sequence**	**Top Hit Accession No.**	**Description of Putative Function**	***E-Value***
Contig_124	XP_002300768.1	60S ribosomal protein L14 [*Populus trichocarpa*]	1.00×10^{-86}
Contig_125	XP_002309519.2	hypothetical protein POPTR_0006s24950g [*Populus trichocarpa*]	3.00×10^{-11}
Contig_126	XP_002325602.2	calmodulin-binding family protein [*Populus trichocarpa*]	1.00×10^{-28}
Contig_127	XP_002307533.2	hypothetical protein POPTR_0005s22170g [*Populus trichocarpa*]	1.00×10^{-105}
Contig_128	XP_006377408.1	hypothetical protein POPTR_0011s05640g [*Populus trichocarpa*]	8.00×10^{-35}
Contig_130	XP_002307493.1	ubiquinol-cytochrome C reductase complex 7.8 kDa family protein [*Populus trichocarpa*]	1.00×10^{-37}
Contig_131	XP_006370905.1	aspartyl protease family protein [*Populus trichocarpa*]	1.00×10^{-109}
Contig_132	XP_002307687.1	thioredoxin h family protein [*Populus trichocarpa*]	2.00×10^{-74}
Contig_133	XP_002315512.1	hypothetical protein POPTR_0010s01590g [*Populus trichocarpa*]	2.00×10^{-20}
Contig_134	XP_002311504.1	hypothetical protein POPTR_0008s12950g [*Populus trichocarpa*]	3.00×10^{-56}
Contig_136	XP_002316233.1	hypothetical protein POPTR_0010s20050g [*Populus trichocarpa*]	3.00×10^{-130}
Contig_137	XP_004161090.1	PREDICTED: ADP-ribosylation factor 1-like [*Cucumis sativus*]	3.00×10^{-66}
Contig_138	XP_002305746.1	60S ribosomal protein L36-3 [*Populus trichocarpa*]	9.00×10^{-60}
Contig_139	XP_002305510.1	putative actin-depolymerizing factor family protein [*Populus trichocarpa*]	4.00×10^{-76}
Contig_141	XP_007209764.1	hypothetical protein PRUPE_ppa013350mg [*Prunus persica*]	2.00×10^{-57}
Contig_146	ADD13529.1	eukaryotic initiation factor 5A hypusine [*Populus trichocarpa* × *Populus deltoides*]	1.00×10^{-08}
Contig_148	XP_007218529.1	hypothetical protein PRUPE_ppa012628mg [*Prunus persica*]	5.00×10^{-82}
Contig_150	XP_006382302.1	hypothetical protein POPTR_0005s00790g [*Populus trichocarpa*]	2.00×10^{-39}
Contig_152	XP_002307243.1	Chain A family protein [*Populus trichocarpa*]	1.00×10^{-113}
Contig_153	XP_006381053.1	C2 domain-containing family protein [*Populus trichocarpa*]	2.00×10^{-103}
Contig_155	XP_006376937.1	hypothetical protein POPTR_0012s11280g [*Populus trichocarpa*]	3.00×10^{-83}
Contig_156	XP_002312531.1	hypothetical protein POPTR_0008s15310g [*Populus trichocarpa*]	3.00×10^{-82}
Contig_157	XP_002301038.1	pollen Ole e 1 allergen and extensin family protein [*Populus trichocarpa*]	2.00×10^{-109}
Contig_158	XP_002312002.1	hypothetical protein POPTR_0008s03670g [*Populus trichocarpa*]	4.00×10^{-68}
Contig_159	ABK93887.1	unknown [*Populus trichocarpa*]	3.00×10^{-79}
Contig_160	XP_002298904.1	calmodulin-binding family protein [*Populus trichocarpa*]	5.00×10^{-94}
Contig_161	XP_009396827.1	PREDICTED: RING-box protein 1a [*Musa acuminata* subsp. *malaccensis*]	1.00×10^{-61}

Table S1. *Cont.*

Sequence	Top Hit Accession No.	Description of Putative Function	*E-Value*
Contig_162	XP_002306334.2	hypothetical protein POPTR_0005s08300g [*Populus trichocarpa*]	9.00×10^{-27}
Contig_163	XP_006374903.1	homeobox-leucine zipper transcription factor family protein [*Populus trichocarpa*]	3.00×10^{-120}
Contig_164	XP_006374052.1	hypothetical protein POPTR_0016s14430g [*Populus trichocarpa*]	2.00×10^{-65}
Contig_165	XP_002321829.1	putative calmodulin-binding family protein [*Populus trichocarpa*]	2.00×10^{-76}
Contig_166	XP_002302758.2	hypothetical protein POPTR_0002s19830g [*Populus trichocarpa*]	3.00×10^{-15}
Contig_167	XP_002321135.1	dienelactone hydrolase family protein [*Populus trichocarpa*]	9.00×10^{-115}
Contig_168	YP_001109494.1	hypothetical protein Poptr_cp015 [*Populus trichocarpa*]	7.00×10^{-11}
Contig_169	XP_002313519.2	fasciclin-like AGP 15 family protein [*Populus trichocarpa*]	2.00×10^{-45}
Contig_170	XP_002318305.1	glutamine synthetase family protein [*Populus trichocarpa*]	1.00×10^{-63}
Contig_171	XP_006381326.1	hypothetical protein POPTR_0006s11810g [*Populus trichocarpa*]	1.00×10^{-75}
Contig_172	XP_006389238.1	hypothetical protein POPTR_0034s00330g [*Populus trichocarpa*]	6.00×10^{-101}
Contig_173	XP_006376549.1	small nuclear ribonucleoprotein U2B [*Populus trichocarpa*]	7.00×10^{-54}
Contig_174	XP_002301486.2	hypothetical protein POPTR_0002s20370g [*Populus trichocarpa*]	1.00×10^{-65}
Contig_176	XP_006375240.1	hypothetical protein POPTR_0014s05560g [*Populus trichocarpa*]	3.00×10^{-06}
Contig_177	XP_002318358.2	hypothetical protein POPTR_0012s01130g [*Populus trichocarpa*]	3.00×10^{-125}
Contig_179	AFY06656.1	actin 2 [*Carica papaya*]	4.00×10^{-82}
Contig_180	XP_006371330.1	Chlorophyll a-b binding protein CP26 [*Populus trichocarpa*]	6.00×10^{-60}
Contig_181	AAD50628.1	α-tubulin [*Gossypium hirsutum*]	2.00×10^{-73}
Contig_183	XP_002318971.1	hypothetical protein POPTR_0013s01340g [*Populus trichocarpa*]	1.00×10^{-35}
Contig_185	XP_006388076.1	hypothetical protein POPTR_0363s00200g [*Populus trichocarpa*]	6.00×10^{-08}
Contig_186	XP_006371993.1	cytidine deaminase 1 family protein [*Populus trichocarpa*]	1.00×10^{-07}
Contig_187	XP_002309570.1	hypothetical protein POPTR_0006s25990g [*Populus trichocarpa*]	4.00×10^{-54}
Contig_188	XP_002310656.1	hypothetical protein POPTR_0007s07820g [*Populus trichocarpa*]	2.00×10^{-42}
Contig_190	XP_002311183.2	Protein phosphatase 2C family protein [*Populus trichocarpa*]	3.00×10^{-26}
Contig_191	XP_002317449.1	hypothetical protein POPTR_0011s10900g [*Populus trichocarpa*]	2.00×10^{-26}
Contig_192	XP_002302195.1	hypothetical protein POPTR_0002s07290g [*Populus trichocarpa*]	1.00×10^{-57}
Contig_197	XP_006423776.1	hypothetical protein CICLE_v10029531mg [*Citrus clementina*]	3.00×10^{-29}

Table S1. *Cont.*

**Sequence**	**Top Hit Accession No.**	**Description of Putative Function**	***E-Value***
Contig_198	XP_002315448.2	hypothetical protein POPTR_0010s24290g [*Populus trichocarpa*]	3.00×10^{-35}
Contig_202	XP_002317034.1	GA family protein [*Populus trichocarpa*]	6.00×10^{-129}
Contig_203	PC4237	trans-cinnamate 4-monooxygenase (EC 1.14.13.11) C − Japanese aspen × large-toothed aspen (fragment) [*Populus sieboldii x Populus grandidentata*]	4.00×10^{-68}
Contig_204	XP_009388207.1	PREDICTED: uncharacterized protein LOC103975022 [*Musa acuminata* subsp. *malaccensis*]	3.00×10^{-21}
Contig_205	XP_002306259.2	dehydration-responsive family protein [*Populus trichocarpa*]	2.00×10^{-43}
Contig_206	XP_002319953.1	Xyloglucan endotransglucosylase/hydrolase protein 9 precursor [*Populus trichocarpa*]	1.00×10^{-164}
Contig_207	XP_006385064.1	hypothetical protein POPTR_0004s23560g [*Populus trichocarpa*]	2.00×10^{-08}
Contig_257	AFR40585.1	cellulase [*Populus nigra*]	2.00×10^{-50}
hs981950.seq	XP_002301930.2	hypothetical protein POPTR_0002s01410g [*Populus trichocarpa*]	3.00×10^{-33}
hs981952.seq	AFW80115.1	hypothetical protein ZEAMMB73_313798 [*Zea mays*]	2.00×10^{-31}
hs981954.seq	XP_002316863.1	14-3-3 brain family protein [*Populus trichocarpa*]	6.00×10^{-124}
hs981955.seq	XP_002303604.1	hypothetical protein POPTR_0003s13150g [*Populus trichocarpa*]	3.00×10^{-46}
hs981956.seq	XP_002317082.2	hypothetical protein POPTR_0011s16080g [*Populus trichocarpa*]	4.00×10^{-24}
hs981958.seq	XP_002298882.1	hypothetical protein POPTR_0001s37970g [*Populus trichocarpa*]	3.00×10^{-48}
hs981964.seq	XP_002317564.1	hypothetical protein POPTR_0011s13600g [*Populus trichocarpa*]	6.00×10^{-119}
hs981965.seq	XP_006376642.1	NADH-dependent glutamate synthase family protein [*Populus trichocarpa*]	2.00×10^{-60}
hs981967.seq	XP_002318034.1	aldehyde dehydrogenase 1 precursor family protein [*Populus trichocarpa*]	7.00×10^{-106}
hs981968.seq	XP_002324923.1	cytochrome b5 domain-containing family protein [*Populus trichocarpa*]	7.00×10^{-60}
hs981969.seq	XP_002314631.1	pyruvate dehydrogenase family protein [*Populus trichocarpa*]	9.00×10^{-119}
hs981973.seq	XP_006383435.1	hypothetical protein POPTR_0005s15480g [*Populus trichocarpa*]	2.00×10^{-61}
hs981977.seq	XP_002324765.1	kinase family protein [*Populus trichocarpa*]	1.00×10^{-126}
hs981979.seq	XP_006376986.1	hypothetical protein POPTR_0012s11830g [*Populus trichocarpa*]	2.00×10^{-110}
hs981982.seq	XP_006382268.1	kinase family protein [*Populus trichocarpa*]	3.00×10^{-111}
hs981984.seq	XP_002308355.1	kinesin motor family protein [*Populus trichocarpa*]	5.00×10^{-108}
hs981986.seq	XP_006381596.1	glycoside hydrolase family 28 family protein [*Populus trichocarpa*]	2.00×10^{-36}
hs981987.seq	XP_002319372.1	secretory carrier membrane family protein [*Populus trichocarpa*]	9.00×10^{-116}

Table S1. *Cont.*

**Sequence**	**Top Hit Accession No.**	**Description of Putative Function**	***E-Value***
hs981993.seq	XP_002312473.1	hypothetical protein POPTR_0008s13600g [*Populus trichocarpa*]	5.00×10^{-106}
hs981994.seq	XP_002315682.2	hypothetical protein POPTR_0010s05330g [*Populus trichocarpa*]	2.00×10^{-92}
hs981995.seq	XP_006382437.1	hypothetical protein POPTR_0005s02150g [*Populus trichocarpa*]	5.00×10^{-13}
hs981998.seq	XP_006376377.1	hypothetical protein POPTR_0013s12480g [*Populus trichocarpa*]	3.00×10^{-99}
hs982000.seq	ABK92698.1	unknown [*Populus trichocarpa*]	2.00×10^{-82}
hs982001.seq	XP_002298474.1	signal peptide peptidase family protein [*Populus trichocarpa*]	1.00×10^{-121}
hs982002.seq	AHB60730.1	3-hydroxyacyl-CoA dehydrogenase [*Populus × canadensis*]	2.00×10^{-111}
hs982003.seq	XP_002319521.1	RALF-LIKE 23 family protein [*Populus trichocarpa*]	2.00×10^{-79}
hs982006.seq	XP_004241245.1	PREDICTED: probable histone H2B.1 [*Solanum lycopersicum*]	1.00×10^{-36}
hs982013.seq	XP_002298619.1	SWIB complex BAF60b domain-containing family protein [*Populus trichocarpa*]	9.00×10^{-91}
hs982014.seq	XP_006368728.1	hypothetical protein POPTR_0001s08140g [*Populus trichocarpa*]	3.00×10^{-68}
hs982018.seq	XP_002318100.1	MA3 domain-containing family protein [*Populus trichocarpa*]	9.00×10^{-92}
hs982021.seq	XP_002323682.2	hypothetical protein POPTR_0016s14580g [*Populus trichocarpa*]	6.00×10^{-106}
hs982022.seq	ABK96230.1	unknown [*Populus trichocarpa × Populus deltoides*]	2.00×10^{-67}
hs982023.seq	XP_002325548.1	hypothetical protein POPTR_0019s11110g [*Populus trichocarpa*]	3.00×10^{-57}
hs982025.seq	XP_002323453.2	myosin heavy chain-related family protein [*Populus trichocarpa*]	7.00×10^{-31}
hs982027.seq	XP_002316205.2	hypothetical protein POPTR_0010s19490g [*Populus trichocarpa*]	3.00×10^{-77}
hs982028.seq	XP_006378814.1	hypothetical protein POPTR_0010s24400g [Populus trichocarpa]	1.00×10^{-45}
hs982031.seq	XP_002277419.1	PREDICTED: DEAD-box ATP-dependent RNA helicase 24 [*Vitis vinifera*]	2.00×10^{-42}
hs982033.seq	XP_006372892.1	hypothetical protein POPTR_0017s06050g [*Populus trichocarpa*]	7.00×10^{-26}
hs982035.seq	XP_002298326.1	hypothetical protein POPTR_0001s25530g [*Populus trichocarpa*]	2.00×10^{-38}
hs982038.seq	XP_002305484.1	hypothetical protein POPTR_0004s17500g [*Populus trichocarpa*]	8.00×10^{-69}
hs982039.seq	XP_006368272.1	hypothetical protein POPTR_0001s01165g [*Populus trichocarpa*]	2.00×10^{-37}
hs982040.seq	ABK93548.1	unknown [*Populus trichocarpa*]	3.00×10^{-16}
hs982041.seq	ACZ67172.1	fasciclin and related adhesion glycoprotein [*Populus nigra*]	5.00×10^{-77}
hs982043.seq	XP_002325735.1	hypothetical protein POPTR_0019s00770g [*Populus trichocarpa*]	5.00×10^{-43}
hs982045.seq	AFZ78611.1	hypothetical protein [*Populus tomentosa*]	2.00×10^{-108}

Table S1. *Cont.*

**Sequence**	**Top Hit Accession No.**	**Description of Putative Function**	***E-Value***
hs982047.seq	XP_002306835.1	lesion inducing family protein [*Populus trichocarpa*]	2.00×10^{-81}
hs982050.seq	XP_006381032.1	hypothetical protein POPTR_0006s05570g [*Populus trichocarpa*]	7.00×10^{-71}
hs982052.seq	XP_006385560.1	hypothetical protein POPTR_0003s08120g [*Populus trichocarpa*]	2.00×10^{-66}
hs982056.seq	XP_002319752.1	hypothetical protein POPTR_0013s06990g [*Populus trichocarpa*]	2.00×10^{-41}
hs982061.seq	XP_002322852.1	leucine-rich repeat family protein [*Populus trichocarpa*]	4.00×10^{-73}
hs982063.seq	XP_006368353.1	Defender against cell death 1 family protein [*Populus trichocarpa*]	3.00×10^{-71}
hs982064.seq	BAN19963.1	tubulin [*Echinochloa phyllopogon*]	1.00×10^{-122}
hs982068.seq	XP_002310849.1	HISTONE H1-3 family protein [*Populus trichocarpa*]	4.00×10^{-64}
hs982069.seq	XP_002308226.1	hypothetical protein POPTR_0006s10280g [*Populus trichocarpa*]	7.00×10^{-42}
hs982070.seq	XP_002319919.2	hypothetical protein POPTR_0013s14200g [*Populus trichocarpa*]	1.00×10^{-79}
hs982071.seq	XP_002315543.1	hypothetical protein POPTR_0010s02830g [*Populus trichocarpa*]	2.00×10^{-72}
hs982073.seq	XP_002322648.1	GCN5-related N-acetyltransferase family protein [*Populus trichocarpa*]	3.00×10^{-29}
hs982074.seq	AGX25216.1	UDP-glucose dehydrogenase [*Populus tomentosa*]	1.00×10^{-119}
hs982076.seq	XP_002306780.1	hypothetical protein POPTR_0005s23280g [*Populus trichocarpa*]	2.00×10^{-94}
hs982079.seq	XP_006372391.1	hypothetical protein POPTR_0017s01160g [*Populus trichocarpa*]	6.00×10^{-20}
hs982080.seq	XP_002322989.2	CAX interacting protein 1 [*Populus trichocarpa*]	2.00×10^{-94}
hs982083.seq	XP_002301862.2	hypothetical protein POPTR_0002s26110g [*Populus trichocarpa*]	1.00×10^{-54}
hs982084.seq	XP_002299610.2	transducin family protein [*Populus trichocarpa*]	9.00×10^{-85}
hs982092.seq	XP_002310189.1	hypothetical protein POPTR_0007s12080g [*Populus trichocarpa*]	9.00×10^{-38}
hs982094.seq	XP_002307436.2	hypothetical protein POPTR_0005s20050g [*Populus trichocarpa*]	3.00×10^{-67}
hs982098.seq	XP_002325223.1	hypothetical protein POPTR_0018s13090g [*Populus trichocarpa*]	2.00×10^{-79}
hs982100.seq	XP_009397606.1	PREDICTED: acyl-CoA-binding protein [*Musa acuminata* subsp. *malaccensis*]	2.00×10^{-42}
hs982110.seq	AAT37950.1	fasciclin-like AGP 7 [*Populus tremula* × *Populus alba*]	2.00×10^{-48}
hs982113.seq	XP_002300147.1	adenosylhomocysteinase family protein [*Populus trichocarpa*]	1.00×10^{-92}
hs982116.seq	XP_002318470.1	UBIQUITIN EXTENSION protein 1 [*Populus trichocarpa*]	2.00×10^{-87}
hs982120.seq	XP_002317708.2	hypothetical protein POPTR_0012s03970g [*Populus trichocarpa*]	2.00×10^{-87}
hs982121.seq	XP_002325099.1	hypothetical protein POPTR_0018s10890g [*Populus trichocarpa*]	4.00×10^{-50}

Table S1. *Cont.*

Sequence	Top Hit Accession No.	Description of Putative Function	*E-Value*
hs982123.seq	XP_002303085.2	kinase-like protein TMKL1 precursor [*Populus trichocarpa*]	1.00×10^{-99}
hs982126.seq	XP_002302318.1	plastocyanin-like domain-containing family protein [*Populus trichocarpa*]	3.00×10^{-33}
hs982128.seq	XP_002303488.2	hypothetical protein POPTR_0003s10630g [*Populus trichocarpa*]	1.00×10^{-114}
hs982129.seq	ABK92634.1	unknown [*Populus trichocarpa*]	3.00×10^{-43}
hs982133.seq	ABK95467.1	unknown [*Populus trichocarpa*]	5.00×10^{-103}
hs982134.seq	XP_002323774.2	myb family transcription factor family protein [*Populus trichocarpa*]	2.00×10^{-102}
hs982138.seq	XP_006376161.1	hypothetical protein POPTR_0013s10370g [*Populus trichocarpa*]	7.00×10^{-15}
hs982142.seq	XP_002308579.1	hypothetical protein POPTR_0006s24880g [*Populus trichocarpa*]	8.00×10^{-92}
hs982144.seq	XP_002315218.1	α-expansin 9 precursor family protein [*Populus trichocarpa*]	3.00×10^{-60}
hs982145.seq	XP_002315088.2	hypothetical protein POPTR_0010s18230g [*Populus trichocarpa*]	8.00×10^{-70}
hs982147.seq	XP_006368701.1	hypothetical protein POPTR_0001s07920g [*Populus trichocarpa*]	5.00×10^{-110}
hs982152.seq	XP_002308357.1	Ubiquitin ligase SINAT2 family protein [*Populus trichocarpa*]	6.00×10^{-37}
hs982153.seq	XP_002308547.2	hypothetical protein POPTR_0006s24250g [*Populus trichocarpa*]	5.00×10^{-30}
hs982154.seq	CAN82657.1	hypothetical protein VITISV_042745 [*Vitis vinifera*]	2.00×10^{-29}
hs982157.seq	XP_007209764.1	hypothetical protein PRUPE_ppa013350mg [*Prunus persica*]	1.00×10^{-52}
hs982160.seq	XP_006384193.1	hypothetical protein POPTR_0004s09830g [*Populus trichocarpa*]	2.00×10^{-86}
hs982176.seq	ACU01854.1	Na^+/H^+ exchanger 3 [*Populus euphratica*]	3.00×10^{-25}
hs982177.seq	XP_002312173.1	outer membrane OMP85 family protein [*Populus trichocarpa*]	3.00×10^{-49}
hs982187.seq	AAT37957.1	fasciclin-like AGP 14 [*Populus tremula* × *Populus alba*]	1.00×10^{-06}
hs982190.seq	ABC70536.1	aquaporin protein [*Vitis pseudoreticulata*]	6.00×10^{-36}
hs982192.seq	XP_002304634.2	hypothetical protein POPTR_0003s15960g [*Populus trichocarpa*]	8.00×10^{-48}
hs982193.seq	XP_002310200.1	RUB1 CONJUGATING ENZYME 1 family protein [*Populus trichocarpa*]	5.00×10^{-48}
hs982199.seq	XP_002302639.2	hypothetical protein POPTR_0002s17390g [*Populus trichocarpa*]	1.00×10^{-28}
hs982200.seq	XP_002312828.2	hypothetical protein POPTR_0009s16370g [*Populus trichocarpa*]	1.00×10^{-96}
hs982202.seq	ABK94630.1	unknown [*Populus trichocarpa*]	8.00×10^{-44}
hs982206.seq	XP_006372862.1	hypothetical protein POPTR_0017s05770g [*Populus trichocarpa*]	3.00×10^{-112}
hs982207.seq	ABF46824.1	putative tubulin α 1 chain [*Fagus sylvatica*]	8.00×10^{-117}

Table S1. *Cont.*

Sequence	Top Hit Accession No.	Description of Putative Function	*E-Value*
hs982209.seq	XP_002309860.1	hypothetical protein POPTR_0007s03140g [*Populus trichocarpa*]	4.00×10^{-25}
hs982210.seq	XP_006384155.1	hypothetical protein POPTR_0004s08540g [*Populus trichocarpa*]	3.00×10^{-15}
hs982211.seq	XP_002300779.1	actin-depolymerizing factor 6 family protein [*Populus trichocarpa*]	5.00×10^{-68}
hs982212.seq	XP_002317362.1	hypothetical protein POPTR_0011s09670g [*Populus trichocarpa*]	1.00×10^{-88}
hs982217.seq	XP_002530842.1	dihydropyrimidinase, putative [*Ricinus communis*]	6.00×10^{-29}
hs982218.seq	XP_002315683.1	hypothetical protein POPTR_0010s05320g [*Populus trichocarpa*]	4.00×10^{-23}
hs982219.seq	XP_002325121.2	hypothetical protein POPTR_0018s11260g [*Populus trichocarpa*]	4.00×10^{-108}
hs982221.seq	AFZ78532.1	annexin [*Populus tomentosa*]	2.00×10^{-50}
hs982222.seq	XP_002304909.1	Peroxidase 42 precursor family protein [*Populus trichocarpa*]	9.00×10^{-110}
hs982223.seq	XP_002300063.1	60S ribosomal protein L27 [*Populus trichocarpa*]	2.00×10^{-77}
hs982224.seq	CAH60720.1	putative plasma membrane intrinsic protein [*Populus tremula* × *Populus tremuloides*]	1.00×10^{-60}
hs982227.seq	BAF01824.1	receptor protein kinase like protein [*Arabidopsis thaliana*]	3.00×10^{-06}
hs982230.seq	XP_006382766.1	DNA-DAMAGE-REPAIR/TOLERATION 2 family protein [*Populus trichocarpa*]	5.00×10^{-107}
hs982231.seq	XP_002303650.1	nucleoside diphosphate kinase family protein [*Populus trichocarpa*]	8.00×10^{-20}
hs982232.seq	XP_006381058.1	mitochondrial import inner membrane translocase subunit Tim17/Tim22/Tim23 family protein [*Populus trichocarpa*]	1.00×10^{-76}
hs982233.seq	XP_002299707.1	Ras-related GTP-binding family protein [*Populus trichocarpa*]	5.00×10^{-94}
hs982235.seq	XP_002304305.1	GTP-binding family protein [*Populus trichocarpa*]	8.00×10^{-83}
hs982236.seq	ABK96222.1	unknown [*Populus trichocarpa x Populus deltoides*]	4.00×10^{-96}
hs982240.seq	XP_002324864.1	clathrin assembly family protein [*Populus trichocarpa*]	5.00×10^{-44}
hs982241.seq	XP_002316195.2	hypothetical protein POPTR_0010s19260g [*Populus trichocarpa*]	3.00×10^{-103}
hs982242.seq	ABK94756.1	unknown [*Populus trichocarpa*]	5.00×10^{-96}
hs982249.seq	XP_002311822.2	hypothetical protein POPTR_0008s20480g [*Populus trichocarpa*]	5.00×10^{-47}
hs982251.seq	XP_002316349.2	hypothetical protein POPTR_0010s22610g [*Populus trichocarpa*]	8.00×10^{-39}
hs982253.seq	XP_010025126.1	PREDICTED: histone H2AX [*Eucalyptus grandis*]	6.00×10^{-47}
hs982261.seq	XP_002311248.1	hypothetical protein POPTR_0008s07330g [*Populus trichocarpa*]	4.00×10^{-109}
hs982264.seq	XP_006386918.1	hypothetical protein POPTR_0002s26090g [*Populus trichocarpa*]	2.00×10^{-18}
hs982265.seq	XP_002298054.1	hypothetical protein POPTR_0001s08970g [*Populus trichocarpa*]	8.00×10^{-84}

Table S1. *Cont.*

Sequence	Top Hit Accession No.	Description of Putative Function	*E-Value*
hs982268.seq	ABK93525.1	unknown [*Populus trichocarpa*]	2.00×10^{-86}
hs982271.seq	XP_002320411.1	hypothetical protein POPTR_0014s13900g [*Populus trichocarpa*]	7.00×10^{-110}
hs982273.seq	ABK94962.1	unknown [*Populus trichocarpa*]	2.00×10^{-103}
hs982278.seq	XP_002488951.1	hypothetical protein SORBIDRAFT_1292s002010 [*Sorghum bicolor*]	2.00×10^{-27}
hs982279.seq	XP_006372560.1	hypothetical protein POPTR_0017s02810g [*Populus trichocarpa*]	1.00×10^{-93}
hs982281.seq	XP_002312129.1	protease inhibitor/seed storage/lipid transfer family protein [*Populus trichocarpa*]	1.00×10^{-73}
hs982283.seq	XP_002312631.2	20S proteasome β subunit family protein [*Populus trichocarpa*]	4.00×10^{-12}
hs982284.seq	ABK94952.1	unknown [*Populus trichocarpa*]	1.00×10^{-73}
hs982285.seq	XP_006369308.1	hypothetical protein POPTR_0001s20900g [*Populus trichocarpa*]	2.00×10^{-74}
hs982286.seq	XP_002307845.2	hypothetical protein POPTR_0006s00420g [*Populus trichocarpa*]	7.00×10^{-96}
hs982287.seq	XP_006372624.1	hypothetical protein POPTR_0017s03345g [*Populus trichocarpa*]	4.00×10^{-21}
hs982288.seq	XP_002321089.2	hypothetical protein POPTR_0014s14370g [*Populus trichocarpa*]	4.00×10^{-78}
hs982289.seq	XP_009117775.1	PREDICTED: wall-associated receptor kinas × 10−like 5 isoform X1 [*Brassica rapa*]	2.00×10^{-10}
hs982294.seq	XP_002308580.1	asparaginyl endopeptidase family protein [*Populus trichocarpa*]	7.00×10^{-41}
hs982301.seq	XP_004155430.1	PREDICTED: uncharacterized protein LOC101232624 [*Cucumis sativus*]	1.00×10^{-64}
hs982303.seq	XP_002318020.1	hypothetical protein POPTR_0012s07820g [*Populus trichocarpa*]	2.00×10^{-42}
hs982307.seq	XP_002303583.2	glutathione peroxidase family protein [*Populus trichocarpa*]	2.00×10^{-64}
hs982308.seq	XP_002298368.2	UDP-GLUCURONIC ACID DECARBOXYLASE family protein [Populus trichocarpa]	1.00×10^{-35}
hs982311.seq	XP_002319131.1	hypothetical protein POPTR_0013s04830g [*Populus trichocarpa*]	6.00×10^{-09}
hs982312.seq	XP_006370577.1	FLAVODOXIN-LIKE QUINONE REDUCTASE 1 family protein [*Populus trichocarpa*]	2.00×10^{-76}
hs982314.seq	ABK92602.1	unknown [*Populus trichocarpa*]	5.00×10^{-47}
hs982316.seq	XP_002310188.1	O2 evolving complex 33kD family protein [*Populus trichocarpa*]	6.00×10^{-81}
hs982318.seq	XP_002303038.1	cytochrome b5 isoform Cb5-C family protein [*Populus trichocarpa*]	8.00×10^{-54}
hs982319.seq	XP_006373560.1	hypothetical protein POPTR_0016s00390g [*Populus trichocarpa*]	8.00×10^{-18}
hs982320.seq	XP_002318013.1	coatomer α subunit-like family protein [*Populus trichocarpa*]	7.00×10^{-102}
hs982325.seq	XP_002314913.2	hypothetical protein POPTR_0010s14710g [*Populus trichocarpa*]	2.00×10^{-22}
hs982328.seq	XP_002313410.1	hypothetical protein POPTR_0009s04410g [*Populus trichocarpa*]	3.00×10^{-24}

Table S1. *Cont.*

Sequence	Top Hit Accession No.	Description of Putative Function	*E-Value*
hs982330.seq	XP_002302357.1	hypothetical protein POPTR_0002s10990g [*Populus trichocarpa*]	2.00×10^{-56}
hs982331.seq	XP_003608708.1	40S ribosomal protein S30 [*Medicago truncatula*]	2.00×10^{-24}
hs982332.seq	EMS49258.1	hypothetical protein TRIUR3_13360 [*Triticum urartu*]	1.00×10^{-05}
hs982333.seq	XP_002302078.2	hypothetical protein POPTR_0002s04630g [*Populus trichocarpa*]	5.00×10^{-106}
hs982334.seq	XP_006370263.1	hypothetical protein POPTR_0001s41100g [*Populus trichocarpa*]	3.00×10^{-42}
hs982335.seq	XP_002318463.1	hypothetical protein POPTR_0012s03000g [*Populus trichocarpa*]	4.00×10^{-29}
hs982337.seq	XP_002317271.1	(2R)-phospho-3-sulfolactate synthas × 10−related family protein [*Populus trichocarpa*]	6.00×10^{-98}
hs982338.seq	XP_006375264.1	t-complex polypeptide 1 family protein [*Populus trichocarpa*]	2.00×10^{-100}
hs982340.seq	XP_006374168.1	hypothetical protein POPTR_0015s03440g [*Populus trichocarpa*]	1.00×10^{-102}
hs982341.seq	XP_002306004.1	Cell division control protein 2 1 [*Populus trichocarpa*]	6.00×10^{-39}
hs982342.seq	KCW51380.1	hypothetical protein EUGRSUZ_J00924 [*Eucalyptus grandis*]	8.00×10^{-44}
hs982344.seq	XP_002302345.2	hypothetical protein POPTR_0002s10640g [*Populus trichocarpa*]	2.00×10^{-19}
hs982345.seq	XP_004136366.1	PREDICTED: plastocyanin, chloroplastic-like [*Cucumis sativus*]	2.00×10^{-62}
hs982346.seq	XP_008063755.1	PREDICTED: ceramide glucosyltransferase-like [*Tarsius syrichta*]	2.10×10^{-5}
hs982347.seq	XP_006381023.1	hypothetical protein POPTR_0006s05480g [*Populus trichocarpa*]	1.00×10^{-19}
hs982348.seq	ABK95361.1	unknown [*Populus trichocarpa*]	2.00×10^{-38}
hs982354.seq	XP_002308982.2	hypothetical protein POPTR_0006s06770g [*Populus trichocarpa*]	2.00×10^{-16}
hs982357.seq	XP_002319496.1	hypothetical protein POPTR_0013s01330g [*Populus trichocarpa*]	2.00×10^{-49}
hs982359.seq	CDX77060.1	BnaC04g38550D [*Brassica napus*]	8.00×10^{-08}
hs982361.seq	XP_006368539.1	hypothetical protein POPTR_0001s04270g [*Populus trichocarpa*]	4.00×10^{-32}
hs982362.seq	XP_002301736.2	hypothetical protein POPTR_0002s24620g [*Populus trichocarpa*]	3.00×10^{-30}
hs982366.seq	XP_002314766.1	hypothetical protein POPTR_0010s11360g [*Populus trichocarpa*]	1.00×10^{-61}
hs982375.seq	XP_002305933.2	hypothetical protein POPTR_0004s07230g [*Populus trichocarpa*]	9.00×10^{-41}
hs982377.seq	XP_002515121.1	conserved hypothetical protein [*Ricinus communis*]	5.00×10^{-81}
hs982387.seq	XP_002302417.1	hypothetical protein POPTR_0002s12100g [*Populus trichocarpa*]	8.00×10^{-24}
hs982388.seq	ABK93109.1	unknown [*Populus trichocarpa*]	1.00×10^{-47}
hs982389.seq	ABK92564.1	unknown [*Populus trichocarpa*]	1.00×10^{-37}

Table S1. *Cont.*

Sequence	Top Hit Accession No.	Description of Putative Function	*E-Value*
hs982392.seq	XP_002310249.2	hypothetical protein POPTR_0007s13110g [*Populus trichocarpa*]	8.00×10^{-105}
hs982394.seq	XP_002309511.2	hypothetical protein POPTR_0006s24820g [*Populus trichocarpa*]	4.00×10^{-09}
hs982396.seq	XP_002320310.2	hypothetical protein POPTR_0014s11730g [*Populus trichocarpa*]	7.00×10^{-99}
hs982397.seq	XP_002323125.2	hypothetical protein POPTR_0016s00870g [*Populus trichocarpa*]	1.00×10^{-80}
hs982400.seq	XP_006387836.1	hypothetical protein POPTR_0531s00200g [*Populus trichocarpa*]	1.00×10^{-19}
hs982401.seq	XP_006373773.1	hypothetical protein POPTR_0016s05270g [*Populus trichocarpa*]	4.00×10^{-21}
hs982402.seq	XP_002305978.2	phosphogluconate dehydrogenase family protein [*Populus trichocarpa*]	5.00×10^{-94}
hs982403.seq	XP_003588326.1	ATP synthase subunit α [*Medicago truncatula*]	3.00×10^{-19}
hs982404.seq	XP_006381262.1	hypothetical protein POPTR_0006s11200g [*Populus trichocarpa*]	7.00×10^{-40}
hs982407.seq	XP_006372103.1	hypothetical protein POPTR_0018s10660g [*Populus trichocarpa*]	1.00×10^{-83}
hs982408.seq	XP_002311641.1	acireductone dioxygenase family protein [*Populus trichocarpa*]	4.00×10^{-74}
hs982409.seq	XP_002304909.1	Peroxidase 42 precursor family protein [*Populus trichocarpa*]	1.00×10^{-15}
hs982410.seq	XP_002299345.1	gene GA family protein [*Populus trichocarpa*]	5.00×10^{-89}
hs982412.seq	ABK94470.1	unknown [*Populus trichocarpa*]	2.00×10^{-13}
hs982413.seq	XP_002325804.2	hypothetical protein POPTR_0019s07200g [*Populus trichocarpa*]	1.00×10^{-61}
hs982415.seq	XP_008465236.1	PREDICTED: magnesium-protoporphyrin IX monomethyl ester [*oxidative*] cyclase, chloroplastic [*Cucumis melo*]	2.00×10^{-17}
hs982418.seq	XP_002324031.1	PYRUVATE DEHYDROGENASE E1 β family protein [*Populus trichocarpa*]	9.00×10^{-57}
hs982420.seq	XP_006379104.1	hypothetical protein POPTR_0009s06900g [*Populus trichocarpa*]	1.00×10^{-28}
hs982423.seq	XP_003615432.1	hypothetical protein MTR_5g067940 [*Medicago truncatula*]	1.00×10^{-11}
hs982424.seq	XP_002305451.2	cysteine proteinase A494 precursor [*Populus trichocarpa*]	9.00×10^{-14}
hs982433.seq	XP_002301263.1	zinc finger family protein [*Populus trichocarpa*]	1.00×10^{-21}
hs982436.seq	ACR36970.1	unknown [*Zea mays*]	6.00×10^{-22}
hs982439.seq	XP_002325838.1	Glutamate dehydrogenase 1 family protein [*Populus trichocarpa*]	2.00×10^{-57}
hs982442.seq	XP_002301105.2	hypothetical protein POPTR_0002s10870g [*Populus trichocarpa*]	5.00×10^{-93}
hs982446.seq	XP_006379920.1	hypothetical protein POPTR_0008s17410g [*Populus trichocarpa*]	1.00×10^{-34}
hs982447.seq	XP_002297937.1	hypothetical protein POPTR_0001s11560g [*Populus trichocarpa*]	2.00×10^{-47}
hs982452.seq	XP_002307247.1	kinase mRNA family protein [*Populus trichocarpa*]	5.00×10^{-05}

Table S1. *Cont.*

Sequence	Top Hit Accession No.	Description of Putative Function	*E-Value*
hs982453.seq	XP_006368524.1	hypothetical protein POPTR_0001s03750g [*Populus trichocarpa*]	9.00×10^{-40}
hs982455.seq	XP_002302216.1	hypothetical protein POPTR_0002s07790g [*Populus trichocarpa*]	1.00×10^{-27}
hs982457.seq	XP_007042563.1	Uncharacterized protein TCM_007167 [*Theobroma cacao*]	1.00×10^{-08}
hs982459.seq	XP_002302765.2	hypothetical protein POPTR_0002s19690g [*Populus trichocarpa*]	2.00×10^{-30}
hs982461.seq	XP_006377936.1	FATTY ACID HYDROXYLASE 1 family protein [*Populustrichocarpa*]	2.00×10^{-26}
hs982462.seq	AAD27823.2	14-3-3 protein [*Populus tremula x Populus alba*]	5.00×10^{-18}
hs982463.seq	ELU14431.1	hypothetical protein CAPTEDRAFT_143212 [*Capitella teleta*]	1.00×10^{-33}
hs982468.seq	XP_002317290.1	hypothetical protein POPTR_0011s02520g [*Populus trichocarpa*]	3.00×10^{-39}
hs982471.seq	XP_002314925.1	hypothetical protein POPTR_0010s15080g [*Populus trichocarpa*]	9.00×10^{-27}
hs982472.seq	XP_002313280.1	60S ribosomal protein L6 [*Populus trichocarpa*]	2.00×10^{-49}
hs982475.seq	XP_002325939.2	hypothetical protein POPTR_0019s10010g [*Populus trichocarpa*]	5.00×10^{-42}
hs982479.seq	XP_002302875.1	autophagy 8d family protein [*Populus trichocarpa*]	1.00×10^{-35}
hs982481.seq	XP_002304544.1	hypothetical protein POPTR_0003s13640g [*Populus trichocarpa*]	1.00×10^{-12}
hs982486.seq	AFZ78532.1	annexin [*Populus tomentosa*]	4.00×10^{-91}
hs982489.seq	XP_002306757.1	40S ribosomal protein S12-1 [*Populus trichocarpa*]	2.00×10^{-11}
hs982490.seq	XP_002320484.1	glycosyl hydrolase family 17 family protein [*Populus trichocarpa*]	8.00×10^{-14}
hs982492.seq	XP_006381160.1	hypothetical protein POPTR_0006s07650g [*Populus trichocarpa*]	4.00×10^{-25}
hs982495.seq	XP_002300529.2	hypothetical protein POPTR_0001s45800g [*Populus trichocarpa*]	6.00×10^{-61}
hs982498.seq	XP_002315591.2	aconitase family protein [*Populus trichocarpa*]	3.00×10^{-03}
hs982499.seq	XP_001177217.2	PREDICTED: histone H3, embryonic-like [S*trongylocentrotus purpuratus*]	6.00×10^{-61}
hs982500.seq	XP_002316593.1	hypothetical protein POPTR_0011s00810g [*Populus trichocarpa*]	7.00×10^{-15}
hs982505.seq	XP_006372062.1	hypothetical protein POPTR_0018s09340g [*Populus trichocarpa*]	2.00×10^{-22}
hs982506.seq	XP_002313301.2	Imidazole glycerol phosphate synthase hisHF family protein [*Populus trichocarpa*]	8.00×10^{-42}
hs982507.seq	XP_006374260.1	nascent polypeptide-associated complex domain-containing family protein [*Populus trichocarpa*]	6.00×10^{-76}
hs982508.seq	XP_002312938.2	hypothetical protein POPTR_0009s14120g [*Populus trichocarpa*]	1.00×10^{-69}
hs982509.seq	XP_002310544.1	hypothetical protein POPTR_0007s04870g [*Populus trichocarpa*]	6.00×10^{-09}
hs982511.seq	XP_002303864.1	hypothetical protein POPTR_0003s18300g [*Populus trichocarpa*]	3.00×10^{-107}

Table S1. *Cont.*

**Sequence**	**Top Hit Accession No.**	**Description of Putative Function**	***E-Value***
hs982512.seq	XP_002324195.1	40S ribosomal protein S3 [*Populus trichocarpa*]	7.00×10^{-18}
hs982513.seq	AFK49226.1	unknown [*Lotus japonicus*]	7.00×10^{-50}
hs982515.seq	XP_006383698.1	basic helix-loop-helix family protein [*Populus trichocarpa*]	1.00×10^{-83}
hs982517.seq	XP_002320627.1	hypothetical protein POPTR_0014s18410g [*Populus trichocarpa*]	2.00×10^{-25}
hs982520.seq	XP_006382051.1	hypothetical protein POPTR_0006s25590g [*Populus trichocarpa*]	5.00×10^{-17}
hs982521.seq	XP_004169868.1	PREDICTED: elongation factor 1-α-like [*Cucumis sativus*]	2.00×10^{-41}
hs982526.seq	XP_002324510.1	UDP-D-GLUCURONATE 4-EPIMERASE 1 family protein [*Populus trichocarpa*]	1.00×10^{-38}
hs982527.seq	XP_002309262.1	hypothetical protein POPTR_0006s21460g [*Populus trichocarpa*]	6.00×10^{-44}
hs982533.seq	XP_002321083.1	hypothetical protein POPTR_0014s14260g [*Populus trichocarpa*]	7.00×10^{-63}
hs982534.seq	XP_002302307.2	hypothetical protein POPTR_0002s09970g [*Populus trichocarpa*]	6.00×10^{-87}
hs982537.seq	XP_002303505.1	chromodomain-helicase-DNA-binding family protein [*Populus trichocarpa*]	2.00×10^{-19}
hs982541.seq	ABK20187.1	cysteine protease inhibitor [*Populus tomentosa*]	3.00×10^{-45}
hs982550.seq	XP_006374574.1	hypothetical protein POPTR_0015s11780g [*Populus trichocarpa*]	3.00×10^{-60}
hs982553.seq	XP_006387123.1	hypothetical protein POPTR_1776s00200g [*Populus trichocarpa*]	4.00×10^{-47}
hs982554.seq	XP_002302417.1	hypothetical protein POPTR_0002s12100g [*Populus trichocarpa*]	5.00×10^{-26}
hs982558.seq	XP_002305967.2	hypothetical protein POPTR_0004s10520g [*Populus trichocarpa*]	1.00×10^{-59}
hs982559.seq	XP_006384895.1	hydroxyproline-rich glycoprotein [*Populus trichocarpa*]	2.00×10^{-23}
hs982562.seq	XP_006378533.1	hypothetical protein POPTR_0010s15260g [*Populus trichocarpa*]	6.00×10^{-47}
hs982563.seq	XP_002316889.1	hypothetical protein POPTR_0011s11790g [*Populus trichocarpa*]	2.00×10^{-07}
hs982564.seq	XP_006372439.1	hypothetical protein POPTR_0017s01640g [*Populus trichocarpa*]	1.00×10^{-34}
hs982566.seq	XP_002318653.2	hypothetical protein POPTR_0012s08370g [*Populus trichocarpa*]	2.00×10^{-41}
hs982574.seq	XP_002303493.2	hypothetical protein POPTR_0003s10680g [*Populus trichocarpa*]	6.00×10^{-67}
hs982580.seq	XP_002314537.2	hypothetical protein POPTR_0010s06940g [*Populus trichocarpa*]	1.00×10^{-05}
hs982581.seq	XP_002307364.1	2-dehydro-3-deoxyphosphoheptonate aldolase family protein [*Populus trichocarpa*]	4.00×10^{-07}
hs982585.seq	XP_002306392.1	hypothetical protein POPTR_0005s09690g [*Populus trichocarpa*]	6.00×10^{-48}
hs982591.seq	XP_002317384.1	60S ribosomal protein L18a-1 [*Populus trichocarpa*]	2.00×10^{-70}
hs982593.seq	ABK93907.1	unknown [*Populus trichocarpa*]	4.00×10^{-28}

Table S1. *Cont.*

Sequence	Top Hit Accession No.	Description of Putative Function	*E-Value*
hs982603.seq	XP_002313663.1	60s acidic ribosomal family protein [*Populus trichocarpa*]	1.00×10^{-13}
hs982606.seq	ABK93345.1	unknown [*Populus trichocarpa*]	7.00×10^{-07}
hs982607.seq	XP_006370970.1	hypothetical protein POPTR_0019s02250g [*Populus trichocarpa*]	5.00×10^{-65}
hs982608.seq	XP_002299789.1	hypothetical protein POPTR_0001s26260g [*Populus trichocarpa*]	4.00×10^{-10}
hs982613.seq	XP_002322888.1	hypothetical protein POPTR_0016s09310g [*Populus trichocarpa*]	6.00×10^{-57}
hs982614.seq	AAF79822.1	T6D22.2 [*Arabidopsis thaliana*]	3.00×10^{-07}
hs982617.seq	XP_006430904.1	hypothetical protein CICLE_v10013116mg [*Citrus clementina*]	1.00×10^{-13}
hs982618.seq	XP_002318959.1	hypothetical protein POPTR_0013s01130g [*Populus trichocarpa*]	3.00×10^{-25}
hs982622.seq	XP_002300939.1	serine carboxypeptidase S10 family protein [*Populus trichocarpa*]	1.00×10^{-36}
hs982626.seq	XP_006370338.1	LIGHT-HARVESTING CHLOROPHYLL BINDING protein 3 [*Populus trichocarpa*]	3.00×10^{-64}
hs982628.seq	EPS74717.1	hypothetical protein M569_00042 [*Genlisea aurea*]	1.00×10^{-16}
hs982630.seq	XP_010100746.1	hypothetical protein L484_005813 [*Morus notabilis*]	7.00×10^{-17}
hs982631.seq	XP_002314650.1	chaperon × 10–related family protein [*Populus trichocarpa*]	2.00×10^{-08}
hs982632.seq	ABK94148.1	unknown [*Populus trichocarpa*]	3.00×10^{-25}
hs982636.seq	XP_002302736.2	kinesin motor family protein [*Populus trichocarpa*]	5.00×10^{-80}
hs982639.seq	XP_002308783.2	hypothetical protein POPTR_0006s01290g [*Populus trichocarpa*]	7.00×10^{-86}
hs982643.seq	XP_006384832.1	SEC1-family transport family protein [*Populus trichocarpa*]	1.00×10^{-84}
hs982644.seq	XP_006384870.1	guanine nucleotide exchange family protein [*Populus trichocarpa*]	1.00×10^{-55}
hs982650.seq	XP_006380079.1	hypothetical protein POPTR_0008s21690g [*Populus trichocarpa*]	3.00×10^{-46}
hs982661.seq	XP_002310999.1	DnaJ family protein [*Populus trichocarpa*]	8.00×10^{-83}
hs982663.seq	XP_002321997.1	calmodulin-binding family protein [*Populus trichocarpa*]	9.00×10^{-31}
hs982665.seq	XP_002308151.1	hypothetical protein POPTR_0006s08390g [*Populus trichocarpa*]	9.00×10^{-16}
hs982667.seq	XP_002298640.1	TCP family transcription factor family protein [*Populus trichocarpa*]	1.00×10^{-18}
hs982669.seq	XP_010048615.1	PREDICTED: protein YIPF6-like [*Eucalyptus grandis*]	2.00×10^{-37}
hs982670.seq	XP_002312280.2	kinesin light chain-related family protein [*Populus trichocarpa*]	2.00×10^{-06}
hs982673.seq	XP_002323472.1	Cell division cycle protein 48 [*Populus trichocarpa*]	6.00×10^{-10}
hs982684.seq	CAB46520.1	putative fructose-bisphosphate aldolase [*Phleum pratense*]	2.00×10^{-09}

Table S1. *Cont.*

**Sequence**	**Top Hit Accession No.**	**Description of Putative Function**	***E-Value***
hs982686.seq	XP_002322910.1	gamma-tonoplast intrinsic protein 2 [*Populus trichocarpa*]	2.00×10^{-25}
hs982691.seq	XP_002303225.2	pectinacetylesterase family protein [*Populus trichocarpa*]	2.00×10^{-17}
hs982692.seq	KGN60302.1	hypothetical protein Csa_3G894500 [*Cucumis sativus*]	4.00×10^{-15}
hs982697.seq	ABK92743.1	unknown [*Populus trichocarpa*]	1.00×10^{-17}
hs982705.seq	XP_006353797.1	PREDICTED: photosystem Q(B) protein-like [*Solanum tuberosum*]	1.00×10^{-67}
hs982709.seq	XP_002325098.2	senescence-associated family protein [*Populus trichocarpa*]	2.00×10^{-66}
hs982716.seq	XP_002489056.1	hypothetical protein SORBIDRAFT_0199s002010 [*Sorghum bicolor*]	1.00×10^{-05}
hs982717.seq	CAD29822.2	putative serine protease [*Populus* × *canadensis*]	2.00×10^{-68}
hs982725.seq	XP_002308400.1	eukaryotic translation initiation factor 5A isoform V family protein [*Populus trichocarpa*]	9.00×10^{-16}
hs982728.seq	XP_006371632.1	hypothetical protein POPTR_0019s13920g [*Populus trichocarpa*]	1.00×10^{-24}
hs982731.seq	XP_004306978.1	PREDICTED: nucleoside diphosphate kinase 1-like [*Fragaria vesca* subsp. *vesca*]	2.00×10^{-13}
hs982736.seq	XP_010325503.1	PREDICTED: mediator of RNA polymerase II transcription subunit 32 [*Solanum lycopersicum*]	3.00×10^{-05}
hs982737.seq	XP_008360709.1	PREDICTED: aquaporin PIP1-3 isoform X2 [*Malus domestica*]	1.00×10^{-06}
hs982738.seq	XP_002313858.2	hypothetical protein POPTR_0009s10300g [*Populus trichocarpa*]	2.00×10^{-16}
hs982743.seq	XP_002313867.2	hypothetical protein POPTR_0009s10020g [*Populus trichocarpa*]	1.00×10^{-19}
hs982746.seq	XP_002322263.1	Protein transport protein SEC61 gamma-3 subunit [*Populus trichocarpa*]	1.00×10^{-28}
hs982747.seq	XP_002316158.1	latex abundant family protein [*Populus trichocarpa*]	7.00×10^{-50}
hs982749.seq	XP_002320334.1	hypothetical protein POPTR_0014s12220g [*Populus trichocarpa*]	9.00×10^{-31}
hs982750.seq	CBI34865.3	unnamed protein product [*Vitis vinifera*]	7.00×10^{-08}
hs982755.seq	XP_002299090.2	hypothetical protein POPTR_0001s03810g [*Populus trichocarpa*]	3.00×10^{-04}
hs982759.seq	XP_006370245.1	hypothetical protein POPTR_0001s40980g [*Populus trichocarpa*]	2.00×10^{-10}
hs982761.seq	XP_002319690.2	hypothetical protein POPTR_0013s05400g [*Populus trichocarpa*]	4.00×10^{-20}
hs982763.seq	XP_004290117.1	PREDICTED: uncharacterized protein LOC101292072 [*Fragaria vesca* subsp. *vesca*]	1.00×10^{-34}
hs982764.seq	XP_002298114.2	hypothetical protein POPTR_0001s17420g [*Populus trichocarpa*]	8.00×10^{-27}
hs982766.seq	XP_002304489.1	glutathione *S*-transferase family protein [*Populus trichocarpa*]	1.00×10^{-75}
hs982774.seq	XP_002299125.2	hypothetical protein POPTR_0001s04570g [*Populus trichocarpa*]	3.00×10^{-46}
hs982776.seq	BAA33801.1	cytosolic phosphoglycerate kinase 1 [*Populus nigra*]	2.00×10^{-65}

Table S1. *Cont.*

**Sequence**	**Top Hit Accession No.**	**Description of Putative Function**	***E-Value***
hs982780.seq	XP_002313736.1	peptidyl-prolyl cis-trans isomerase family protein [*Populus trichocarpa*]	8.00×10^{-13}
hs982781.seq	XP_002305699.1	20S proteasome β subunit B family protein [*Populus trichocarpa*]	3.00×10^{-70}
hs982788.seq	XP_006374602.1	hypothetical protein POPTR_0015s12580g [*Populus trichocarpa*]	1.00×10^{-05}
hs982792.seq	XP_002309632.2	hypothetical protein POPTR_0006s27100g [*Populus trichocarpa*]	4.00×10^{-07}
hs982796.seq	ABK94287.1	unknown [*Populus trichocarpa*]	1.00×10^{-34}
hs982804.seq	XP_006381415.1	hypothetical protein POPTR_0006s12660g [*Populus trichocarpa*]	1.00×10^{-13}
hs982809.seq	XP_006375200.1	40S ribosomal protein S11 [*Populus trichocarpa*]	3.00×10^{-12}
hs982818.seq	XP_002312968.2	hypothetical protein POPTR_0009s01630g [*Populus trichocarpa*]	2.00×10^{-08}
hs982825.seq	3DM0_A	Chain A, Maltose Binding Protein Fusion With Rack1 From A. Thaliana	6.00×10^{-05}
hs982831.seq	XP_006387793.1	hypothetical protein POPTR_0575s00200g [*Populus trichocarpa*]	1.00×10^{-07}
hs982836.seq	XP_006368940.1	hypothetical protein POPTR_0001s150802g [*Populus trichocarpa*]	1.00×10^{-60}
hs982837.seq	Q9LDQ7.1	RecName: Full = *S*-adenosylmethionine synthase; Short = AdoMet synthase; AltName: Full = Methionine adenosyltransferase; Short = MAT [*Camellia sinensis*]	2.00×10^{-07}
hs982844.seq	XP_002317013.2	hypothetical protein POPTR_0011s14470g [*Populus trichocarpa*]	2.00×10^{-23}
hs982846.seq	XP_002322910.1	gamma-tonoplast intrinsic protein 2 [*Populus trichocarpa*]	3.00×10^{-72}

Table S2. KEGG annotation and its corresponding EC number of *L. gmelinii* 634 unigenes.

Pathway	Seqs in Pathway	Enzyme	Ezyme ID	Seqs of Enzyme	Seqs	Pathway ID
Aminobenzoate degradation	1	hydratase	ec:4.2.1.17	1	hs982002.seq	map00627
Naphthalene degradation	1	dehydrogenase	ec:1.1.1.1	1	Contig_192	map00626
Flavone and flavonol biosynthesis	1	3-*O*-methyltransferase	ec:2.1.1.76	1	Contig_69	map00944
Flavone and flavonol biosynthesis	1	entry	ec:2.1.1.149	1	Contig_69	map00944
Fluorobenzoate degradation	1	maleylacetate enol-lactonase	ec:3.1.1.45	1	Contig_167	map00364
Benzoate degradation	1	hydratase	ec:4.2.1.17	1	hs982002.seq	map00362
Chlorocyclohexane and chlorobenzene degradation	1	maleylacetate enol-lactonase	ec:3.1.1.45	1	Contig_167	map00361
Biosynthesis of ansamycins	1	glycolaldehydetransferase	ec:2.2.1.1	1	hs982418.seq	map01051
Steroid biosynthesis	1	monooxygenase	ec:1.14.13.72	1	Contig_127	map00100
Pantothenate and CoA biosynthesis	1	hydantoinase	ec:3.5.2.2	1	hs982217.seq	map00770
Caprolactam degradation	1	hydratase	ec:4.2.1.17	1	hs982002.seq	map00930
Caprolactam degradation	1	dehydrogenase	ec:1.1.1.35	1	hs982002.seq	map00930
Tyrosine metabolism	1	dehydrogenase	ec:1.1.1.1	1	Contig_192	map00350
Fatty acid elongation	1	hydratase	ec:4.2.1.17	1	hs982002.seq	map00062
Fatty acid elongation	1	dehydrogenase	ec:1.1.1.35	1	hs982002.seq	map00062
Biosynthesis of unsaturated fatty acids	1	hydratase	ec:4.2.1.17	1	hs982002.seq	map01040
Geraniol degradation	1	hydratase	ec:4.2.1.17	1	hs982002.seq	map00281
Geraniol degradation	1	dehydrogenase	ec:1.1.1.35	1	hs982002.seq	map00281
Primary bile acid biosynthesis	1	dehydrogenase	ec:1.1.1.35	1	hs982002.seq	map00120
D-Glutamine and D-glutamate metabolism	1	dehydrogenase [NAD(P)+]	ec:1.4.1.3	1	hs982439.seq	map00471
Drug metabolism - cytochrome P450	1	dehydrogenase	ec:1.1.1.1	1	Contig_192	map00982
Histidine metabolism	1	dehydrogenase (NAD+)	ec:1.2.1.3	1	hs981967.seq	map00340
Galactose metabolism	1	uridylyltransferase	ec:2.7.7.9	1	Contig_109	map00052
Metabolism of xenobiotics by cytochrome P450	1	dehydrogenase	ec:1.1.1.1	1	Contig_192	map00980
Glycerolipid metabolism	1	dehydrogenase (NAD+)	ec:1.2.1.3	1	hs981967.seq	map00561
α-Linolenic acid metabolism	1	hydratase	ec:4.2.1.17	1	hs982002.seq	map00592

Table S2. *Cont.*

Pathway	Seqs in Pathway	Enzyme	Ezyme ID	Seqs of Enzyme	Seqs	Pathway ID
Linoleic acid metabolism	1	acid dehydrogenase	ec:1.14.99.33	1	hs982314.seq	map00591
Arachidonic acid metabolism	1	peroxidase	ec:1.11.1.9	1	hs982307.seq	map00590
Cyanoamino acid metabolism	2	gentiobiase	ec:3.2.1.21	1	hs982396.seq	map00460
Cyanoamino acid metabolism	2	hydroxymethyltransferase	ec:2.1.2.1	1	hs982330.seq	map00460
Chloroalkane and chloroalkene degradation	2	dehydrogenase	ec:1.1.1.1	1	Contig_192	map00625
Chloroalkane and chloroalkene degradation	2	dehydrogenase (NAD+)	ec:1.2.1.3	1	hs981967.seq	map00625
Toluene degradation	2	maleylacetate enol-lactonase	ec:3.1.1.45	1	Contig_167	map00623
Toluene degradation	2	dehydrogenase	ec:1.1.1.35	1	hs982002.seq	map00623
Selenocompound metabolism	2	*S*-methyltransferase	ec:2.1.1.14	2	Contig_82, Contig_83	map00450
Selenocompound metabolism	2	synthase	ec:2.1.1.13	2	Contig_82, Contig_83	map00450
Limonene and pinene degradation	2	hydratase	ec:4.2.1.17	1	hs982002.seq	map00903
Limonene and pinene degradation	2	dehydrogenase (NAD+)	ec:1.2.1.3	1	hs981967.seq	map00903
Glutathione metabolism	2	dehydrogenase (NADP+-dependent, decarboxylating)	ec:1.1.1.44	1	hs982402.seq	map00480
Glutathione metabolism	2	peroxidase	ec:1.11.1.9	1	hs982307.seq	map00480
T cell receptor signaling pathway	2	protein-tyrosine kinase	ec:2.7.10.2	1	hs982366.seq	map04660
T cell receptor signaling pathway	2	phosphatase	ec:3.1.3.16	1	hs982142.seq	map04660
Propanoate metabolism	2	hydratase	ec:4.2.1.17	1	hs982002.seq	map00640
Propanoate metabolism	2	dehydrogenase (NAD+)	ec:1.2.1.3	1	hs981967.seq	map00640
Tryptophan metabolism	2	hydratase	ec:4.2.1.17	1	hs982002.seq	map00380
Tryptophan metabolism	2	dehydrogenase	ec:1.1.1.35	1	hs982002.seq	map00380
Tryptophan metabolism	2	dehydrogenase (NAD+)	ec:1.2.1.3	1	hs981967.seq	map00380
Retinol metabolism	2	dehydrogenase	ec:1.1.1.1	1	Contig_192	map00830
Retinol metabolism	2	dehydrogenase	ec:1.2.1.36	1	hs981967.seq	map00830

Table S2. *Cont.*

Pathway	Seqs in Pathway	Enzyme	Ezyme ID	Seqs of Enzyme	Seqs	Pathway ID
Valine, leucine and isoleucine degradation	2	hydratase	ec:4.2.1.17	1	hs982002.seq	map00280
Valine, leucine and isoleucine degradation	2	dehydrogenase	ec:1.1.1.35	1	hs982002.seq	map00280
Valine, leucine and isoleucine degradation	2	dehydrogenase (NAD+)	ec:1.2.1.3	1	hs981967.seq	map00280
Drug metabolism - other enzymes	2	ali-esterase	ec:3.1.1.1	1	Contig_152	map00983
Drug metabolism - other enzymes	2	hydantoinase	ec:3.5.2.2	1	hs982217.seq	map00983
Glyoxylate and dicarboxylate metabolism	2	ligase	ec:6.3.1.2	1	Contig_170	map00630
Glyoxylate and dicarboxylate metabolism	2	hydroxymethyltransferase	ec:2.1.2.1	1	hs982330.seq	map00630
Citrate cycle (TCA cycle)	2	dehydrogenase (acetyl-transferring)	ec:1.2.4.1	2	hs981969.seq, hs982418.seq	map00020
Fructose and mannose metabolism	2	aldolase	ec:4.1.2.13	1	Contig_113	map00051
Fructose and mannose metabolism	2	fructokinase (phosphorylating)	ec:2.7.1.4	1	hs982284.seq	map00051
Carbon fixation pathways in prokaryotes	2	reductase [NAD(P)H]	ec:1.5.1.20	1	hs982129.seq	map00720
Carbon fixation pathways in prokaryotes	2	dehydrogenase	ec:1.1.1.35	1	hs982002.seq	map00720
Stilbenoid, diarylheptanoid and gingerol biosynthesis	3	4-monooxygenase	ec:1.14.13.11	1	Contig_203	map00945
Stilbenoid, diarylheptanoid and gingerol biosynthesis	3	O-methyltransferase	ec:2.1.1.104	2	Contig_112, hs981993.seq	map00945
Flavonoid biosynthesis	3	4-monooxygenase	ec:1.14.13.11	1	Contig_203	map00941
Flavonoid biosynthesis	3	O-methyltransferase	ec:2.1.1.104	2	Contig_112, hs981993.seq	map00941
Pyruvate metabolism	3	dehydrogenase (acetyl-transferring)	ec:1.2.4.1	2	hs981969.seq, hs982418.seq	map00620
Pyruvate metabolism	3	dehydrogenase (NAD+)	ec:1.2.1.3	1	hs981967.seq	map00620
Butanoate metabolism	3	hydratase	ec:4.2.1.17	1	hs982002.seq	map00650
Butanoate metabolism	3	epimerase	ec:5.1.2.3	1	hs982002.seq	map00650
Butanoate metabolism	3	dehydrogenase	ec:1.1.1.35	1	hs982002.seq	map00650

Table S2. *Cont.*

Pathway	Seqs in Pathway	Enzyme	Ezyme ID	Seqs of Enzyme	Seqs	Pathway ID
Butanoate metabolism	3	dehydrogenase (acetyl-transferring)	ec:1.2.4.1	2	hs981969.seq, hs982418.seq	map00650
Fatty acid metabolism	3	dehydrogenase	ec:1.1.1.1	1	Contig_192	map00071
Fatty acid metabolism	3	hydratase	ec:4.2.1.17	1	hs982002.seq	map00071
Fatty acid metabolism	3	epimerase	ec:5.1.2.3	1	hs982002.seq	map00071
Fatty acid metabolism	3	dehydrogenase	ec:1.1.1.35	1	hs982002.seq	map00071
Fatty acid metabolism	3	dehydrogenase (NAD+)	ec:1.2.1.3	1	hs981967.seq	map00071
Purine metabolism	3	RNA polymerase	ec:2.7.7.6	1	hs981998.seq	map00230
Purine metabolism	3	kinase	ec:2.7.4.6	2	hs982231.seq, hs982731.seq	map00230
Glycine, serine and threonine metabolism	3	dehydrogenase	ec:1.1.1.1	1	Contig_192	map00260
Glycine, serine and threonine metabolism	3	mutase	ec:5.4.2.1	1	hs982348.seq	map00260
Glycine, serine and threonine metabolism	3	hydroxymethyltransferase	ec:2.1.2.1	1	hs982330.seq	map00260
Carbon fixation in photosynthetic organisms	3	aldolase	ec:4.1.2.13	1	Contig_113	map00710
Carbon fixation in photosynthetic organisms	3	glycolaldehydetransferase	ec:2.2.1.1	1	hs982418.seq	map00710
Carbon fixation in photosynthetic organisms	3	kinase	ec:2.7.2.3	1	hs982776.seq	map00710
Porphyrin and chlorophyll metabolism	3	ferro-protoporphyrin chelatase	ec:4.99.1.1	1	hs982394.seq	map00860
Porphyrin and chlorophyll metabolism	3	reductase	ec:1.2.1.70	1	hs982442.seq	map00860
Porphyrin and chlorophyll metabolism	3	IX monomethyl ester (oxidative) cyclase	ec:1.14.13.81	1	hs982415.seq	map00860
β-Alanine metabolism	3	hydratase	ec:4.2.1.17	1	hs982002.seq	map00410
β-Alanine metabolism	3	dehydrogenase (NAD+)	ec:1.2.1.3	1	hs981967.seq	map00410
β-Alanine metabolism	3	hydantoinase	ec:3.5.2.2	1	hs982217.seq	map00410
Ascorbate and aldarate metabolism	3	6-dehydrogenase	ec:1.1.1.22	1	hs982074.seq	map00053
Ascorbate and aldarate metabolism	3	oxidase	ec:1.10.3.3	1	Contig_136	map00053
Ascorbate and aldarate metabolism	3	dehydrogenase (NAD+)	ec:1.2.1.3	1	hs981967.seq	map00053
Arginine and proline metabolism	4	lyase	ec:4.3.2.1	1	hs982344.seq	map00330

Table S2. *Cont.*

**Pathway**	**Seqs in Pathway**	**Enzyme**	**Ezyme ID**	**Seqs of Enzyme**	**Seqs**	**Pathway ID**
Arginine and proline metabolism	4	dehydrogenase [NAD(P)+]	ec:1.4.1.3	1	hs982439.seq	map00330
Arginine and proline metabolism	4	ligase	ec:6.3.1.2	1	Contig_170	map00330
Arginine and proline metabolism	4	dehydrogenase (NAD+)	ec:1.2.1.3	1	hs981967.seq	map00330
Oxidative phosphorylation	4	oxidase	ec:1.9.3.1	1	hs982121.seq	map00190
Oxidative phosphorylation	4	reductase	ec:1.10.2.2	1	Contig_130	map00190
Oxidative phosphorylation	4	diphosphatase	ec:3.6.1.1	2	Contig_51, hs982392.seq	map00190
One carbon pool by folate	4	reductase [NAD(P)H]	ec:1.5.1.20	1	hs982129.seq	map00670
One carbon pool by folate	4	synthase	ec:2.1.1.13	2	Contig_82, Contig_83	map00670
One carbon pool by folate	4	hydroxymethyltransferase	ec:2.1.2.1	1	hs982330.seq	map00670
Alanine, aspartate and glutamate metabolism	4	synthase (NADH)	ec:1.4.1.14	1	hs981965.seq	map00250
Alanine, aspartate and glutamate metabolism	4	lyase	ec:4.3.2.1	1	hs982344.seq	map00250
Alanine, aspartate and glutamate metabolism	4	dehydrogenase [NAD(P)+]	ec:1.4.1.3	1	hs982439.seq	map00250
Alanine, aspartate and glutamate metabolism	4	ligase	ec:6.3.1.2	1	Contig_170	map00250
Lysine degradation	4	hydratase	ec:4.2.1.17	1	hs982002.seq	map00310
Lysine degradation	4	dehydrogenase	ec:1.1.1.35	1	hs982002.seq	map00310
Lysine degradation	4	*N*-methyltransferase	ec:2.1.1.43	2	Contig_198, hs982092.seq	map00310
Lysine degradation	4	dehydrogenase (NAD+)	ec:1.2.1.3	1	hs981967.seq	map00310
Pyrimidine metabolism	4	RNA polymerase	ec:2.7.7.6	1	hs981998.seq	map00240
Pyrimidine metabolism	4	kinase	ec:2.7.4.6	2	hs982231.seq, hs982731.seq	map00240
Pyrimidine metabolism	4	hydantoinase	ec:3.5.2.2	1	hs982217.seq	map00240
Pentose phosphate pathway	5	aldolase	ec:4.1.2.13	1	Contig_113	map00030
Pentose phosphate pathway	5	deoxyribokinase	ec:2.7.1.15	2	hs982206.seq, hs982284.seq	map00030

Table S2. *Cont.*

Pathway	Seqs in Pathway	Enzyme	Ezyme ID	Seqs of Enzyme	Seqs	Pathway ID
Pentose phosphate pathway	5	dehydrogenase (NADP+-dependent, decarboxylating)	ec:1.1.1.44	1	hs982402.seq	map00030
Pentose phosphate pathway	5	glycolaldehydetransferase	ec:2.2.1.1	1	hs982418.seq	map00030
Nitrogen metabolism	6	synthase (NADH)	ec:1.4.1.14	1	hs981965.seq	map00910
Nitrogen metabolism	6	dehydrogenase [NAD(P)+]	ec:1.4.1.3	1	hs982439.seq	map00910
Nitrogen metabolism	6	oxidase	ec:1.9.3.1	1	hs982121.seq	map00910
Nitrogen metabolism	6	reductase	ec:1.10.2.2	1	Contig_130	map00910
Nitrogen metabolism	6	ligase	ec:6.3.1.2	1	Contig_170	map00910
Nitrogen metabolism	6	reductase (NADH)	ec:1.7.1.1	1	hs982318.seq	map00910
Cysteine and methionine metabolism	6	adenosyltransferase	ec:2.5.1.6	1	Contig_100	map00270
Cysteine and methionine metabolism	6	*S*-methyltransferase	ec:2.1.1.14	2	Contig_82, Contig_83	map00270
Cysteine and methionine metabolism	6	synthase	ec:2.1.1.13	2	Contig_82, Contig_83	map00270
Cysteine and methionine metabolism	6	*S*-adenosylhomocysteine synthase	ec:3.3.1.1	1	hs982113.seq	map00270
Cysteine and methionine metabolism	6	dioxygenase [iron(II)-requiring]	ec:1.13.11.54	1	hs982408.seq	map00270
Cysteine and methionine metabolism	6	oxidase	ec:1.14.17.4	1	Contig_93	map00270
Pentose and glucuronate interconversions	7	6-dehydrogenase	ec:1.1.1.22	1	hs982074.seq	map00040
Pentose and glucuronate interconversions	7	uridylyltransferase	ec:2.7.7.9	1	Contig_109	map00040
Pentose and glucuronate interconversions	7	pectin demethoxylase	ec:3.1.1.11	3	Contig_174, hs982265.seq, hs982796.seq	map00040
Pentose and glucuronate interconversions	7	pectin depolymerase	ec:3.2.1.15	1	hs981986.seq	map00040
Pentose and glucuronate interconversions	7	dehydrogenase (NAD+)	ec:1.2.1.3	1	hs981967.seq	map00040
Phenylalanine metabolism	7	4-monooxygenase	ec:1.14.13.11	1	Contig_203	map00360
Phenylalanine metabolism	7	hydratase	ec:4.2.1.17	1	hs982002.seq	map00360

Table S2. *Cont.*

Pathway	Seqs in Pathway	Enzyme	Ezyme ID	Seqs of Enzyme	Seqs	Pathway ID
Phenylalanine metabolism	7	lactoperoxidase	ec:1.11.1.7	3	hs982221.seq, hs982222.seq, hs982409.seq	map00360
Phenylalanine metabolism	7	*O*-methyltransferase	ec:2.1.1.104	2	Contig_112, hs981993.seq	map00360
Amino sugar and nucleotide sugar metabolism	7	4-α-galacturonosyltransferase	ec:2.4.1.43	1	hs981964.seq	map00520
Amino sugar and nucleotide sugar metabolism	7	6-dehydrogenase	ec:1.1.1.22	1	hs982074.seq	map00520
Amino sugar and nucleotide sugar metabolism	7	uridylyltransferase	ec:2.7.7.9	1	Contig_109	map00520
Amino sugar and nucleotide sugar metabolism	7	decarboxylase	ec:4.1.1.35	1	hs982308.seq	map00520
Amino sugar and nucleotide sugar metabolism	7	fructokinase (phosphorylating)	ec:2.7.1.4	1	hs982284.seq	map00520
Amino sugar and nucleotide sugar metabolism	7	chitodextrinase	ec:3.2.1.14	2	Contig_110, hs982288.seq	map00520
Phenylpropanoid biosynthesis	8	4-monooxygenase	ec:1.14.13.11	1	Contig_203	map00940
Phenylpropanoid biosynthesis	8	*O*-methyltransferase	ec:2.1.1.68	1	Contig_69	map00940
Phenylpropanoid biosynthesis	8	lactoperoxidase	ec:1.11.1.7	3	hs982221.seq, hs982222.seq, hs982409.seq	map00940
Phenylpropanoid biosynthesis	8	*O*-methyltransferase	ec:2.1.1.104	2	Contig_112, hs981993.seq	map00940
Phenylpropanoid biosynthesis	8	gentiobiase	ec:3.2.1.21	1	hs982396.seq	map00940
Glycolysis/Gluconeogenesis	8	dehydrogenase	ec:1.1.1.1	1	Contig_192	map00010
Glycolysis/Gluconeogenesis	8	aldolase	ec:4.1.2.13	1	Contig_113	map00010
Glycolysis/Gluconeogenesis	8	mutase	ec:5.4.2.1	1	hs982348.seq	map00010
Glycolysis/Gluconeogenesis	8	kinase	ec:2.7.2.3	1	hs982776.seq	map00010
Glycolysis/Gluconeogenesis	8	dehydrogenase (acetyl-transferring)	ec:1.2.4.1	2	hs981969.seq, hs982418.seq	map00010
Glycolysis/Gluconeogenesis	8	dehydrogenase (phosphorylating)	ec:1.2.1.12	1	hs982236.seq	map00010

Table S2. *Cont.*

Pathway	Seqs in Pathway	Enzyme	Ezyme ID	Seqs of Enzyme	Seqs	Pathway ID
Glycolysis/Gluconeogenesis	8	dehydrogenase (NAD+)	ec:1.2.1.3	1	hs981967.seq	map00010
Methane metabolism	8	aldolase	ec:4.1.2.13	1	Contig_113	map00680
Methane metabolism	8	lactoperoxidase	ec:1.11.1.7	3	hs982221.seq, hs982222.seq, hs982409.seq	map00680
Methane metabolism	8	synthase	ec:4.4.1.19	1	hs982337.seq	map00680
Methane metabolism	8	mutase	ec:5.4.2.1	1	hs982348.seq	map00680
Methane metabolism	8	hydroxymethyltransferase	ec:2.1.2.1	1	hs982330.seq	map00680
Methane metabolism	8	dehydrogenase	ec:1.1.1.284	1	Contig_192	map00680
Starch and sucrose metabolism	14	4-α-galacturonosyltransferase	ec:2.4.1.43	1	hs981964.seq	map00500
Starch and sucrose metabolism	14	endo-1,3-β-D-glucosidase	ec:3.2.1.39	1	hs982286.seq	map00500
Starch and sucrose metabolism	14	endo-1,4-β-D-glucanase	ec:3.2.1.4	1	Contig_257	map00500
Starch and sucrose metabolism	14	synthase (GDP-forming)	ec:2.4.1.29	1	Contig_10	map00500
Starch and sucrose metabolism	14	6-dehydrogenase	ec:1.1.1.22	1	hs982074.seq	map00500
Starch and sucrose metabolism	14	uridylyltransferase	ec:2.7.7.9	1	Contig_109	map00500
Starch and sucrose metabolism	14	gentiobiase	ec:3.2.1.21	1	hs982396.seq	map00500
Starch and sucrose metabolism	14	pectin demethoxylase	ec:3.1.1.11	3	Contig_174, hs982265.seq, hs982796.seq	map00500
Starch and sucrose metabolism	14	synthase (UDP-forming)	ec:2.4.1.12	2	Contig_10, Contig_171	map00500
Starch and sucrose metabolism	14	decarboxylase	ec:4.1.1.35	1	hs982308.seq	map00500
Starch and sucrose metabolism	14	fructokinase (phosphorylating)	ec:2.7.1.4	1	hs982284.seq	map00500
Starch and sucrose metabolism	14	pectin depolymerase	ec:3.2.1.15	1	hs981986.seq	map00500
